# Impaired reinforcement learning and coding of prediction errors in patients with cerebellar degeneration - a study with EEG and voxel-based morphometry

**DOI:** 10.3758/s13415-025-01303-2

**Published:** 2025-05-28

**Authors:** Adam M. Berlijn, Dana M. Huvermann, Eric Bechler, Andreas Thieme, Alfons Schnitzler, Christian Bellebaum, Dagmar Timmann, Martina Minnerop, Jutta Peterburs

**Affiliations:** 1 Faculty of Mathematics and Natural Sciences, Heinrich Heine University Düsseldorf, Düsseldorf, Germany; 2 Institute of Clinical Neuroscience and Medical Psychology, Medical Faculty & University Hospital Düsseldorf, Heinrich Heine University Düsseldorf, Düsseldorf, Germany; 3 Institute of Neuroscience and Medicine (INM-1), Research Centre Jülich, Jülich, Germany; 4 Department of Neurology and Center for Translational and Behavioral Neurosciences (C-TNBS), Essen University Hospital, University of Duisburg-Essen, Essen, Germany; 5 Core Facility for Magnetic Resonance Imaging, Medical Faculty and University Hospital Düsseldorf, Heinrich Heine University Düsseldorf, Düsseldorf, Germany; 6 Department of Neurology, Center for Movement Disorders and Neuromodulation, Medical Faculty & University Hospital Düsseldorf, Heinrich Heine University Düsseldorf, Düsseldorf, Germany; 7 Institute of Systems Medicine and Department of Human Medicine, MSH Medical School Hamburg, Hamburg, Germany

**Keywords:** Reinforcement learning, Reward prediction errors, Performance monitoring, Cerebellum, Neurodegeneration, Ataxia

## Abstract

This study investigated cerebellar involvement in reinforcement learning and prediction error (RL-PE) processing. Participants with pure cerebellar degeneration and demographically matched healthy controls performed a probabilistic feedback-based learning task while brain activity was recorded using electroencephalography (EEG). Structural magnetic resonance imaging was used to quantify cerebellar gray matter volume (GMV). Data from 21 cerebellar and 25 control participants were included in the analysis. We aimed to determine if feedback-based learning was impaired in patients relative to controls, and if single-trial RL-PEs were reflected in FRN, P3a, and P3b in the event-related potential (ERP) in patients and controls. Analysis of behavioral data revealed no differences in accuracy between patients and controls. Crucially, ERP analysis revealed that, while in controls, coding of RL-PEs was found in FRN and P3a for positive and in P3b for positive and negative feedback, these effects were absent in patients. Voxel-based morphometry revealed widely distributed cerebellar GMV reduction in patients, most pronounced in bilateral Crus I/ II and bilateral lobules I-IV. Multiple regressions in patients revealed a negative correlation between GMV in bilateral Crus I and II and FRN amplitudes. The present study extends previous evidence for cerebellar involvement in RL-PE processing in humans and advances our understanding of the cerebellum’s role in performance monitoring and adaptive control of behavior.

**Supplementary Information:**

The online version contains supplementary material available at 10.3758/s13415-025-01303-2.

## Introduction

Reinforcement learning is a key cognitive ability that enables humans to process performance-related external feedback and to adapt their decisions and actions accordingly (Ullsperger et al., 2014). Central to reinforcement learning is the processing of reward prediction errors (RL-PEs) which arise when an action is followed by an unexpected reward/punishment or by omission of an expected reward/punishment. Reinforcement learning has been shown to rely on a distributed network of cortical and subcortical cerebral structures, such as midbrain/striatum, the medial prefrontal cortex, and the anterior cingulate cortex (ACC: for a review, see Ullsperger et al., 2014). Interestingly, recent findings also point to a prominent role of the cerebellum in reinforcement learning and particularly the processing of RL-PEs. For instance, recent rodent studies showed reward sensitivity in several cerebellar cell populations such as climbing fibers (Ohmae & Medina, 2015) and the mossy fiber-granule cell pathway (Wagner et al., 2017, for a review see Kostadinov & Häusser, 2022). In humans, several functional magnetic resonance imaging (fMRI) studies showed cerebellar activations during feedback-based learning (e.g., Peterburs et al., 2018; for a comprehensive review, see Berlijn, Huvermann, Schneider et al., 2024b). In addition, a meta-analysis on reward anticipation and reward outcome processing (Kruithof et al., 2023) revealed functional connectivity between the cerebellum and higher order, associative brain regions like the rostral ACC, also consistent with the notion of cerebellar involvement in RL-PE processing.

Several previous studies have pointed to alterations of feedback-processing in patients with cerebellar lesions. For instance, Thoma et al. (2008) reported impaired reversal learning in patients with cerebellar stroke. Rustemeier et al. (2016) and Huvermann et al. (2025) recorded electroencephalography (EEG) while patients with cerebellar lesions and healthy controls performed a feedback-based learning task. Both studies revealed differences in patients compared to healthy controls in components of the event-related potential (ERP) that can be seen as indices of feedback processing, e.g., the feedback-related negativity (FRN, Holroyd & Coles, 2002; Nieuwenhuis et al., 2004) and the P300 (Polich, 2007). This is in line with studies reporting altered error processing in patients with cerebellar stroke or cerebellar degeneration (e.g., Peterburs et al., 2012, 2015; Tunc et al., 2019), given that error processing is functionally linked to feedback processing (Bellebaum & Colosio, 2014; Peterburs & Desmond, 2016).

The FRN is a negative deflection in the ERP that peaks approximately 200–350 ms after feedback onset (Gehring & Willoughby, 2002; Holroyd & Coles, 2002). Of note, a positive deflection within the same time window of the feedback-locked ERP has been identified to be sensitive to rewards and has been termed reward positivity (RewP, Proudfit, 2015). The RewP appears to reflect a positive RL-PE and appears to be preceded by a negative deflection (around 200 ms, therefore also termed N200). Since we were interested in the fundamental influence of feedback valence and RL-PE on the feedback-locked ERP, we opted to use the term FRN, consistent with previous studies (e.g., Rustemeier et al., 2016; Huvermann et al., 2025). The FRN has been shown to be sensitive to feedback valence (Gehring & Willoughby, 2002; Nieuwenhuis et al., 2005; Pfabigan et al., 2011), and to reflect RL-PEs during learning (Fischer & Ullsperger, 2013; Burnside et al., 2019; Weber & Bellebaum, 2024). Furthermore, the FRN is sensitive to feedback timing (Faßbender et al., 2023; Peterburs et al., 2016; Weber & Bellebaum, 2024). FRN amplitude differences between negative and positive feedback typically decrease with increasing delay between response and feedback (Peterburs et al., 2016), consistent with a shift away from striatal processing for delayed compared to immediate feedback (Foerde & Shohamy, 2011). The FRN amplitude itself increases with increasing feedback delay (Peterburs et al., 2016). In line with the latter finding, Weber and Bellebaum (2024) found more negative amplitudes for delayed compared to immediate feedback using a single-trial analysis approach.

Another ERP component linked to feedback processing is the P300, a positive deflection in the ERP peaking between 300 and 500 ms after stimulus onset (Polich, 2007). While findings concerning effects of feedback valence on the P300 are mixed (see Ullsperger, 2024 for a review), the P300 is sensitive to feedback expectancy (Pfabigan et al., 2011; Rustemeier et al., 2016; Walentowska et al., 2019). Indeed, two subcomponents of the P3, the frontocentral P3a and the centroparietal P3b, were found to be sensitive to RL-PE coding (Fischer & Ullsperger, 2013; Hoy et al., 2021; Ullsperger, 2024; Weber & Bellebaum, 2024; Wessel & Huber, 2019).

Regarding alterations of feedback processing in patients with cerebellar lesions, the findings by Rustemeier et al. (2016) revealed enhanced differentiation of positive and negative feedback as reflected in the negative-positive difference signal in the FRN time window in patients compared to controls, possibly indicative of altered coding of RL-PEs. However, RL-PEs were not explicitly modelled in this study. In the study by Huvermann et al. (2025), coding of RL-PEs in the FRN was directly investigated and modelled, first in patients with cerebellar lesions compared to controls, and second in a complementary experiment using single-pulse transcranial magnetic stimulation (TMS) applied to the left posterolateral cerebellum or a control site (vertex) in healthy subjects. Results showed a lack of RL-PE coding in the FRN in cerebellar lesion patients compared to controls, and for cerebellar compared to vertex TMS.

The present study aimed to further characterize the cerebellum’s role in reinforcement learning by investigating patients with progressive cerebellar degeneration, and by focusing on coding of RL-PEs in the feedback-locked ERP during feedback learning as a function of feedback timing. To this end, feedback in the probabilistic learning task was presented either immediately (500 ms post-response) or with a 6500 ms delay. Of note, we deviated from the preregistration (see below) by using a single-trial analysis approach with modelling of RL-PE values for each trial (Weber & Bellebaum, 2024), and by assessment of choice switching (Huvermann et al., 2025).

With regard to behavior, given the functional link between error and feedback processing (Bellebaum & Colosio, 2014) and previous reports of impaired error processing in patients with cerebellar degeneration (e.g., Peterburs et al., 2015), we hypothesized that patients would show decreased accuracy relative to healthy controls. This deficit in accuracy in patients was expected to be altered by feedback timing. Using single-trial data, we hypothesized reduced choice switching in patients compared to controls, consistent with impaired reversal learning and thus behavioral flexibility (Thoma et al., 2008). Regarding neural responses, we expected deficient/absent coding of the RL-PE in the FRN in patients compared to controls for immediate feedback. We additionally analyzed the P3a and P3b, expecting to find expectancy effects, i.e., differences between trials with high unsigned RL-PEs (= low expectancy) compared to low unsigned RL-PEs (= high expectancy).

Last, we investigated whether specific cerebellar subregions could be linked to potential alterations in behavior or neural response patterns in patients by analyzing cerebellar gray may volume (GMV) using whole-brain and cerebellar voxel-based morphometry (VBM). Based on the cerebellar functional topography (King et al., 2019) and previous findings on error processing (Peterburs et al., 2015), posterolateral cerebellar regions were hypothesized to be most critical.

The study protocol and hypotheses were preregistered on the Open Science Framework (OSF: https://osf.io/fgw8h/)

## Methods

### Sample

Fifty-nine participants were recruited, of which 28 were patients and 31 healthy controls. Information on the a priori power analysis for the preregistered repeated measures ANOVA is provided in the supplement. For the patient group, only individuals with pure forms of cerebellar degeneration were included, such as spinocerebellar ataxia type 6 (SCA6), for details see Table 1.

**Table 1:** Patient characterization

Number	Type of disease	Age (years)	Sex	EHI – LQ
sub-pat-01	SCA6	54	m	100
sub-pat-03	SCAR8	29	m	100
sub-pat-04	SCA6	66	f	100
sub-pat-05	SCA14	64	m	73.33
sub-pat-06	SCA48	38	m	100
sub-pat-08	SCA27B	29	m	100
sub-pat-09	SCA27B	70	f	100
sub-pat-10	SCA14	65	f	100
sub-pat-13	SCA14	43	m	100
sub-pat-14	SCA14	40	m	100
sub-pat-16	SCA14	61	m	100
sub-pat-17	CACNA1 A	55	m	100
sub-pat-18	SCA14	38	f	100
sub-pat-19	SCA27B	67	m	100
sub-pat-20	SCA14	62	f	100
sub-pat-22	SCA6	71	m	100
sub-pat-23	SCAR10	32	f	100
sub-pat-24	SCAR10 (ANO10)	33	f	100
sub-pat-26	SCA6	66	m	100
sub-pat-27	Early-onset cerebellar ataxia^1^	43	m	100
sub-pat-28	SCA14*	53	f	20

Note. SCA = Spinocerebellar ataxia (autosomal dominant), SCAR10 = Spinocerebellar ataxia - autosomal recessive, CACNA1 A = calcium voltage-gated channel subunit alpha1 A mutation, m = male, f = female

^1^Genetic defect not yet found

^*^Patient did not take part in the MRI session. Handedness was measured using the EHI obtaining the lateralization quotient (LQ)

Patients were recruited from the ataxia clinics of the Departments of Neurology at the University Hospitals Düsseldorf and Essen, Germany. Exclusion criteria for patients were alcohol and illicit substance abuse, presence of other neurological disorders or psychiatric disorders except for mild depression. As participants received structural MRI, typical exclusion criteria for MRI studies applied, such as prosthesis, metallic clips, pacemakers, insulin pumps, claustrophobia, and pregnancy. All patients underwent neurological and neuropsychological assessment (for details, see Table 2 and Table S1 in the supplement). Healthy participants were recruited via newspaper advertisements and postings at the respective university and/or clinic. Control subjects were matched to the patients regarding sex, age, and educational attainment. Exclusion criteria for control subjects were presence or history of any neurological disorders, psychiatric disorders other than sufficiently treated depression (e.g., antidepressants/psychotherapy; this was due to the high prevalence of depression in the patients), and alcohol or illicit substance abuse. In addition, MRI exclusion criteria also applied. All control participants underwent neuropsychological testing but did not receive a neurological examination.

**Table 2:** Group means (*M*) and standard deviations (*SD*) for scores obtained in the neurological and neuropsychological assessment

Function and test	Patients (*M/SD)*	Controls (*M*/*SD*)	*p*-value
Intelligence quotient (MWT-B)	108/10.81	111.4/9.84	.282
Severity of ataxia (SARA)	9.17/3.47	NA	
Neuropsychological deficits (CCAS-Scale)	1.33/1.46	1.48/1.76	.945
Depressed mood (BDI-II)	8.38/5.73	3.12/2.82	<.001

*Note*. *t*-tests for parametric and Wilcoxon rank test for non-parametric distribution were calculated. *N* = 21 patients, *N* = 25 controls

After inspecting the structural MRI data (T1- and T2-weighted scans; not available for one patient and one control subject) and EEG data as well as evaluating the questionnaires, a total of thirteen participants had to be excluded from data analyses (seven patients, six controls). One patient and one control subject were excluded due to severe white matter hyperintensities/lesions as rated by three reviewers (A.B., A.R., M.M.) including an experienced neurologist (M.M.) using the Scale for Age-Related White Matter Changes (ARWMC, scoring with 3, Wahlund et al., 2001). Two individuals from the control group (hydrocephalus, lacunar lesion within the cerebellum) and one patient (hydrocephalus) were excluded based on incidental findings. One patient and one control subject were excluded due to current psychological disorders (major depression and agoraphobia, respectively). Inspecting the EEG data, another six participants (four patients and two control subjects) had to be excluded due to poor signal quality (excessive noise due to technical problems) which did not allow pooling and reconstructing the electrodes of interest FCz and Pz (*n* = 3), a wrongly selected EEG sampling rate (*n* = 1), excessive movements during the experimental task (*n* = 1), and data loss due to a technical problem (*n* = 1).

In total, data from 21 patients (*n* = 8 female, mean age in years = 51.38, *SD* = 14.70) and 25 healthy controls (*n* = 10 female, mean age in years 52.52, *SD* = 13.72) were included in the behavioral and ERP analyses. In this sample, age (*t*(41.48) = 0.27, *p* =.789) and education years (*t*(43.70) = 1.44, *p* =.156) did not differ between groups. VBM was performed using a subset of *n* = 18 patients because one patient (sub-pat-28) had not been able to participate in the MRI session, and two patients with SCAR10 (sub-pat-23, sub-pat-24) had massive atrophy of the cerebellum and were identified in a homogeneity analysis on cerebellar gray matter volume as extreme outliers (see Figure S1 for a boxplot, and Figure S2 for a gray matter slice for each patient in the supplement). Detailed demographic information about each included patient can be found in Table 1. For the group comparison, a subset of *n* = 24 control subjects was used because MRI data were not available for one individual.

The present study was conducted in accordance with the ethical principles for medical research involving human subjects outlined in the revised version of the Declaration of Helsinki (World Medical Association, 2013), and had received ethical clearance by the Ethics Committees of the Faculty of Medicine at Heinrich Heine University Düsseldorf, Germany, and of the University Hospital Essen, Germany.

### Neurological and neuropsychological assessment

Severity of ataxia symptoms in patients was assessed using the Scale for the Assessment and Rating of Ataxia (SARA; Schmitz-Hübsch et al., 2006). To assess possible cognitive and/or affective impairments, the German version (Thieme et al., 2020) of the Cerebellar Cognitive Affective Syndrome Scale (CCAS; Hoche et al., 2018) was used in both groups. In addition, the intelligence quotient (IQ) was estimated based on performance in a multiple-choice vocabulary test, i.e., the MWT-B (*Mehrfachwahl-Wortschatz-Intelligenztest Version B*; Lehrl et al., 1995). The BDI-II (Beck Depression Inventory 2; Beck et al., 1996) was used to measure severity of depression, and handedness was assessed using the EHI (Edinburgh Handedness Inventory: Oldfield, 1971). Group means and comparisons for the different tests and questionnaires are provided in Table 2. Table S1 in the supplement contains further neurological scores and results from questionnaires on motor and nonmotor symptoms as well as general quality of life.

### Task

Participants completed two versions of a probabilistic feedback-based learning task as described by Eppinger et al. (2008), Bellebaum and Colosio (2014), and Huvermann et al. (2025) in two sessions that took place on two consecutive days. EEG was recorded concurrently. The task versions differed in feedback timing and stimulus sets (see below) but were otherwise identical.

**Fig. 1:**
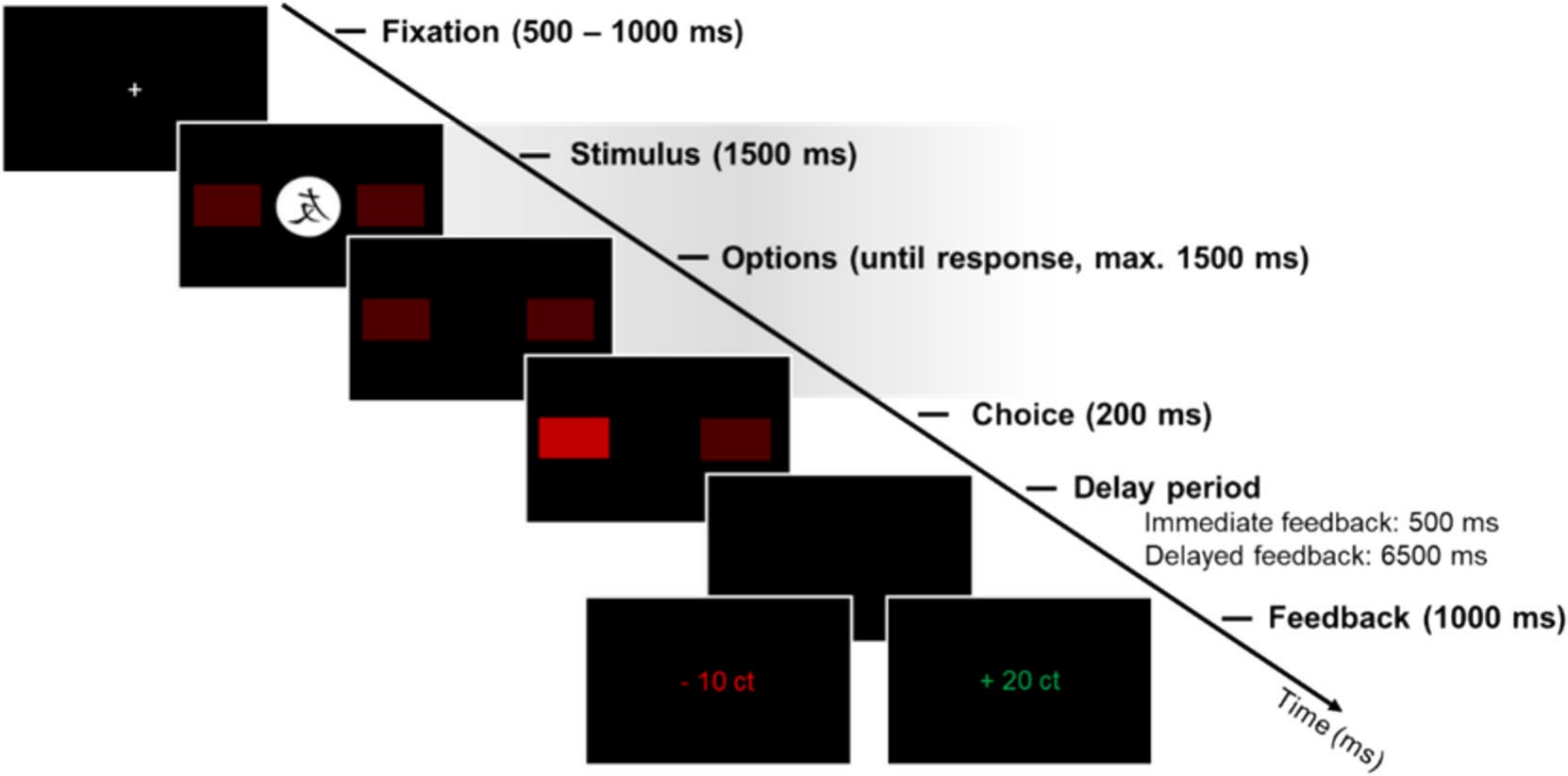
Schematic illustration of the time course and sequence of stimulus presentation in one trial of the probabilistic feedback-based learning task. Each trial started with a fixation cross, followed by a stimulus along with two response options (left or right) presented for 1500 ms. Responses had to be made within 3000 ms after stimulus onset as indicated by the gray shading. The choice was highlighted on screen for 200 ms. Subsequently, feedback was provided after a delay period of either 500 ms (immediate feedback) or 6500 ms (delayed feedback), with positive feedback indicated by “+ 20 ct” in green color and negative feedback with “−10 ct” in red color. Feedback was displayed for 1000 ms

The task consisted of eight blocks with 40 trials each, thus 320 trials in total. Figure 1 illustrates the time course and sequence of stimulus presentation in one trial of the task. Each trial began with a fixation cross presented for 500–1500 ms. Next, one of four abstract stimuli was presented for 1500 ms, and participants were asked to respond by pressing the left or right button on a response box. The choice options were represented by red rectangles which stayed on screen for further 1500 ms, if no response was given. Once a response was given, the respective rectangle was highlighted for 200 ms, followed by a black screen for 500 ms in the task version with immediate feedback condition, and for 6500 ms in the task version with delayed feedback. Last, feedback was displayed for 1000 ms. Feedback was either displayed as a monetary reward of"+20ct"in green font as positive feedback or"−10ct"in red font as negative feedback. Two stimuli were linked to random feedback (50 % positive and 50 % negative feedback independent of response) and served as distractors, while for the other two stimuli, choosing the correct option (left or right, respectively), resulted in positive feedback 90% of the time and in negative feedback 10% of the time. These two stimuli will henceforth be referred to as “learnable”.

In case a participant had learned so fast that they exceeded the learning criterion of 65 % correct responses for learnable stimuli by the second of eight blocks, a new stimulus set was provided to increase the number of pre-learning trials. This was the case in 32 participants (15 patients, 17 controls). If a participant did not exceed the learning criterion until the eighth and last block, a ninth block was added to generate post-learning trials. This was the case in one patient and one control subject for one task version. Trials with responses made within 100 ms after stimulus onset, responses given later than 3000 ms after stimulus onset, or multiple responses were excluded from analysis.

### Procedure

Participants were seated in a brightly lit room in front of a laptop (DELL® Precision M4800, 15.4 inch with a resolution of 1920 x 1080 pixels and a refresh rate of 60 Hz). Left and right button presses were made using a response box (Cedrus RB-740, Science Plus Group, Groningen, NL) placed in front of the laptop. A third key was used to navigate through instruction slides and pauses. Across both sessions, the distance between response box and laptop was kept constant. After positioning the participant, the EEG cap was fitted, and the electrodes were prepared. Subsequently, standardized task instructions were given, and five practice trials were presented before the first block of the experiment started. Following the completion of the probabilistic feedback-based learning task (approx. 30 min for the immediate feedback version, or 60 minutes for the delayed feedback version), demographic data, neuropsychological and neurological testing and MRI data were obtained. The entire test session on the first day took approx. 2.5–3 hours. On the second day, the other version of the probabilistic feedback-based learning task was conducted, either with immediate or delayed feedback and a different stimulus set to avoid any spill-over effects between the sessions. Version order and stimulus set were balanced across participants. The test session on the second day took approx. 1.5–2 hours.

### EEG acquisition and preprocessing

EEG was recorded from 28 active Ag/AgCl electrodes on an actiCAP (BrainProducts GmbH, Munich, Germany) with the following electrode sites: Fz, F3, F4, F7, F8, FC1, FC2, FC5, FC6, Cz, C3, C4, CP1, CP2, CP5, CP6, T7, T8, Pz, P3, P4, P7, P8, PO9, PO10, O1, O2, Oz. FCz served as on-line reference, and AFz was used as ground electrode. Both mastoids were recorded for later re-referencing. Horizontal (hEOG) eye movements were measured with an electrode positioned next to the outer canthus of the left eye, and vertical (vEOG) eye movements/blinks were recorded using electrode position Fp1, respectively. BrainVision Recorder software (version 1.21; BrainProducts, Munich, Germany) was used for recording. Data were amplified with a BrainAmp DC amplifier, and impedances were kept below 25 kΩ. Data were sampled at 1000 Hz**.**

First, the EEG signal in each data set was visually inspected for noisy electrodes which were removed before re-referencing. On average, 2.93 (*SD* = 1.33) electrodes (mostly occipital) had to be removed in eleven participants. The signal was then re-referenced to the mean of the mastoid electrodes so that FCz could be restored as an active electrode. Direct current (DC) detrending and a Butterworth filter with a low cut-off of 0.1 Hz (time constant: 1.59), a high cut-off of 30 Hz, and a notch filter of 50 Hz were applied. As a next step, ocular correction independent component analysis as implemented in BrainVision Analyzer 2 (version 2.2, Brain Products GmbH, Gilching, Germany) was applied using hEOG and vEOG. Data were then segmented into epochs of 800 ms, starting 200 ms before and ending 600 ms after feedback onset. Next, baseline correction was applied based on the 200 ms preceding feedback onset, and automatic artifact rejection was performed. Here, segments with a voltage step above 50 µV/ms, values over 100 µV or below −100 µV, a difference of more than 100 µV between values, or an activity lower than 0.1 µV within an interval of 100 ms were excluded. On average, *M* = 6.53 % (*SD* = 9.28 %) feedback-locked segments were rejected per participant. Last, data were exported via generic data export and then imported into MATLAB (version R2020b: MathWorks, Natick, Massachusetts, USA) to run custom scripts to further process ERP components at single-trial level.

We extracted FRN amplitudes from the single-trial ERP based on FRN latency in the average ERP per person for each condition: FRN latency was defined as the latency of the local maximum negative peak within the time window from 200 to 350 ms post-feedback at FCz (Bellebaum & Colosio, 2014; Bellebaum et al., 2010; Peterburs et al., 2016). FRN amplitudes were determined based on the mean signal in a time window between 20 ms before to 20 after the peak (40 ms length for averaging: Huvermann et al., 2025). If no peak was detected in the respective average, the trials in the condition were coded as outliers. For P3a and P3b (scored at FCz and Pz, respectively, Huvermann et al., 2025; Kirschner et al., 2024), mean amplitudes in the time window from 300 to 500 ms after feedback onset were used.

### Prediction error modelling

A reinforcement learning model was used to estimate the prediction error δ associated with positive and negative feedback in each trial (PE: Sutton & Barto, 2018). Many previous studies have used this approach (e.g., Bray & O'Doherty, 2007; Chase et al., 2011; Ichikawa et al., 2010; McDougle et al., 2018; Pessiglione et al., 2006). The action values *Q* and PE δ were modelled using a Rescorla-Wagner model (Rescorla & Wagner, 1972). For the estimation of the PE δ, the information from the individual trial including the received feedback *R* and the given response $a$ of each participant were used:

$$\begin{array}{l}{Q}_{a,t+1}= {Q}_{a,t}+ \alpha \times {\delta }_{t}\\ {\delta }_{t}= {R}_{a,t} -{Q}_{a,t}\end{array}$$

A softmax function (Sutton & Barto, 2018) was used to model response probabilities by estimating the probability of the chosen action and its respective action value *Q* for each action option *a* and time point *t* (trial):

$${p}_{{a}_{1},t}= \frac{{e}^{\beta \times {Q}_{{a}_{1},t}}}{{e}^{\beta \times {Q}_{{a}_{1},t}}+{e}^{\beta \times {Q}_{{a}_{2},t}}}$$

The model was fitted using the fmincon function implemented in MATLAB (version R2020b). This function minimized the negative sum of log-likelihoods minus a gamma distribution of β with a shape parameter of 2 and scale parameter of 3 to adjust for high β (Leong et al., 2017; McDougle et al., 2018). The learning rate α was separately estimated for positive and negative feedback and each stimulus. We allowed α to assume any value between 0 and 1. In addition, we calculated an inverse temperature β for exploration behavior which could assume any value between 0 and 50. In the statistical analysis, valence and the unsigned PE were used as separate predictors, as the signed PE correlates with feedback valence.

### Voxel-based morphometry

Imaging data were acquired with a 3 T MR scanner (MAGNETOM Trio, a Tim System, Siemens Healthineers AG, Forchheim, Germany) using a 12-channel head coil. This included 3D T1-weighted magnetisation-prepared rapid acquisition gradient-echo (MPRAGE) sequence (voxel size 1 mm^3^). The complete MRI protocol can be found in the OSF folder. DICOM files were transformed into the Brain Imaging Data Format (BIDS: Gorgolewski et al., 2017) by using the *BIDSmapper* and *BIDScoiner* applications (Zwiers et al., 2022).

VBM (Ashburner & Friston, 2000, 2005) was used to characterize GMV loss in patients relative to controls, and to relate possible group differences found in the feedback-based learning task and/or in EEG measures to specific cerebellar regions using multiple regression. For whole-brain VBM, we used the Computational Anatomy Toolbox (CAT12: Gaser et al., 2022) implemented in the Statistical Parametric Mapping software package (SPM12: Wellcome Department of Cognitive Neurology, London, UK) in MATLAB (version R2020b). The default preprocessing procedure was used, and we calculated the total intracranial volume (TIV) for each participant. In addition, we checked the homogeneity of the whole-brain data for all participants. Last, the preprocessed gray matter images were smoothed using an 8 mm full-width half-maximum (FWHM) gaussian kernel.

For cerebellar VBM, we applied an optimized approach to isolate the cerebellum using the Spatially Unbiased Infratentorial toolbox (SUIT: Diedrichsen, 2006). We followed previous analysis protocols to conduct VBM in SUIT (Burciu et al., 2013; Peterburs et al., 2015) and visually inspected the preprocessed images for each subsequent analysis step to ensure sufficient data quality. First, the cerebellum and brainstem were isolated using the standard isolation and segmentation procedure in SUIT which created gray and white matter maps as well as the respective masks.

For six datasets, we additionally used T2-weighted images (Sampling Perfection with Application optimized Contrasts using different flip angle Evolution: SPACE) and fluid attenuated inversion recovery (FLAIR; see supplemental material for MRI protocol details) to optimize the isolation and segmentation procedure of the cerebellum due to poor results after visual inspection of an initial isolation and segmentation run. The T1-weighted images were oriented according to the AC-PC line, and the T2-weighted images were subsequently registered on the reoriented T1-weighted images. After optimizing these six datasets, results improved. In the next step, all cerebellar masks were hand-corrected by an expert (B.B.) using MRIcron (https://www.nitrc.org/projects/mricron). This step was conducted to correct the automatically generated masks for any occipital cortex within the cerebellar mask and to add any missing cerebellar matter. Afterwards, the isolated and segmented gray matter maps were spatially normalized to the SUIT template using the normalization procedure with Dartel. Next, we resliced the spatially normalized gray matter maps using Dartel into SUIT-space with 1 mm^3^ voxel size and with a 2 mm FWHM gaussian kernel.

### Statistical analysis

We deviated from the preregistration and conducted mixed linear model (MLM) analysis instead of analysis of variance (ANOVA) because we decided to analyze the RL-PE which is a single-trial predictor and cannot be analyzed using ANOVA. MLMs are robust against missing values and can additionally model each participant as a random factor to explain more variance. MLMs were conducted in R (R Core Team, version 4.0.3) using RStudio (version 1.3.959) and the lme4 package (version: 1.1.25, Bates et al., 2015). Meteyard and Davies (2020) advise in their best practice guidelines to use the maximum model including all within-subject main and interaction effects as random effects as long as no errors in model fit occur (e.g., convergence errors or singular fits). The *buildmer* (version 2.8) package was used to find the maximum model by fitting the MLM in an ordered stepwise manner by deleting terms that led to convergence errors. In addition, the optimizer was changed from default to bobyqa when the buildmer model did not converge after using the *lmer* function to check the model. Outlier detection was conducted using Cook’s distance.

For the behavioral data, accuracy was calculated as the mean percentage of correct responses for all learnable trials per block corrected for misses (> 3000 ms), multiple responses, and too fast responding (within 100 ms following stimulus onset). The between-subjects factor group (patients, controls) and the within-subject factors feedback timing (immediate, delayed) and block (block 1–8, scaled using the built-in *scale* function) were included as fixed-effects and the within-subject factors main effects and interaction as random slopes per participant:

$$\text{Accuracy }\sim \text{ group}\times \text{feedback timing}\times \text{block}+ \left(1+\text{feedback timing}\times \left.\text{block}\right|\text{participant}\right)$$

To investigate behavioral flexibility, choice switching was calculated on the single-trial level by checking whether the response in the next trial with the same stimulus was the same or different compared to the current trial. Choice switching was analyzed with the factors group, feedback valence, feedback timing, response type, and block (block 1–8, scaled), and the within-subject factors were again used as random slopes per participant

$$\text{Choice switching }\sim \text{ group}\times \text{feedback timing}\times \text{feedback valence}\times \text{response type}\times \text{block}+ \left(1+\text{feedback timing}+\left.\text{feedback valence}+\text{response type}+\text{feedback valence}:\text{response type}\right|\text{participant}\right)$$

For the single-trial EEG analyses, separate models were calculated for FRN, P3a, and P3b amplitudes as dependent variables. We calculated the unsigned prediction error (unsigned PE) using the unsigned value of each PE to separate the sign from the PE and subtracting the value from 0.5 to center the range (−0.5 minimum and 0.5 maximum value). The between-subjects factor group (patient, control) and the categorical within-subject factors feedback timing (immediate, delayed), feedback valence (positive, negative), and learnability (learnable, unlearnable) were included. In addition, we modelled the continuous predictor unsigned PE. Their main effects and interactions were used as fixed effects. To account for individual differences, a random intercept per participant and random slopes per participant for all within-subject factors main and interaction effects were used:

$$\begin{array}{l}\text{FRN }\sim \text{ group}\times \text{feedback timing}\times \text{unsigned PE}\times \text{feedback valence}\times \text{learnability}+\left(1+\text{feedback timing}\times \left.\text{feedback valence}+\text{learnability}+\text{feedback valence : learnability + feedback timing : learnability}\right|\text{participant}\right)\\ \text{P}3\text{a }\sim \text{ group}\times \text{feedback timing}\times \text{unsigned PE}\times \text{feedback valence}\times \text{ learnability}+\left(1+\text{feedback timing}+ \left.\text{feedback valence}\right|\text{participant}\right)\\ \text{P}3\text{b }\sim \text{ group}\times \text{feedback timing}\times \text{unsigned PE}\times \text{feedback valence}\times \text{ learnability}+\left(1+\text{feedback timing}+\left.\text{feedback valence}+\text{unsigned PE}+\text{feedback timing}: \text{unsigned PE}\right|\text{participant}\right)\end{array}$$

All categorical predictors were simple coded: group (0.5 = patient, −0.5 = control), feedback timing (0.5 = delayed feedback, −0.5 = immediate feedback), feedback valence (0.5 = positive feedback, −0.5 = negative feedback), learnability (0.5 = learnable, −0.5 = unlearnable), response type (0.5 = correct, −0.5 = false). The *lmerTest* package (version: 3.1.3, Kuznetsova et al., 2017) in R including the Satterthwaite’s method to estimate the degrees of freedom and to generate *p*-values for MLMs was used. *P*-values below .05 were considered as statistically significant. Interactions were resolved using the *probe_interaction* function to estimate simple slopes based on the moderating factors of interest. Total numbers of included trials for each condition grouped by the factors used in the MLM are provided in the supplement (see Table S13).

The preprocessed whole-brain volumes and cerebellar gray matter volumes were analyzed using two-sample *t*-tests for group comparisons. For patients only, cerebellar GMV was correlated (separately for positive and negative correlations) with parameters derived from the learning task that yielded significant group differences. TIV and age were used as covariates of no interest for all analyses within the framework of the general linear model (GLM) as implemented in SPM12. First, we compared the GMV for the whole-brain data between patients and controls (contrast control > patient) using two-sample *t*-test. Second, we used the cerebellar GMV for the same contrast. Third, for multiple regression analysis, we aggregated the single-trial FRN across all trials for each patient as a covariate of interest. All regressors were demeaned before entering the final model. For the statistical threshold, we used the Family-wise error (FWE) corrected *p*-value <.05 for the between-subjects comparison and an uncorrected *p*-value (*p* <.001) for the multiple regression. Last, the contrasts were masked using the SUIT atlas with 1 mm resolution, and the cerebellar lobules were labelled using the probabilistic MRI atlas of the human cerebellum according to Diedrichsen et al. (2009).

## Results

Since this study’s focus was on potential differences between patients with cerebellar degeneration and healthy controls regarding feedback learning and RL-PE processing, we only report significant effects that included the group factor or replicated known effects from the literature in the main text. For complete statistical results, readers are kindly referred to the respective results tables provided in the supplement.

### Accuracy

**Fig. 2:**
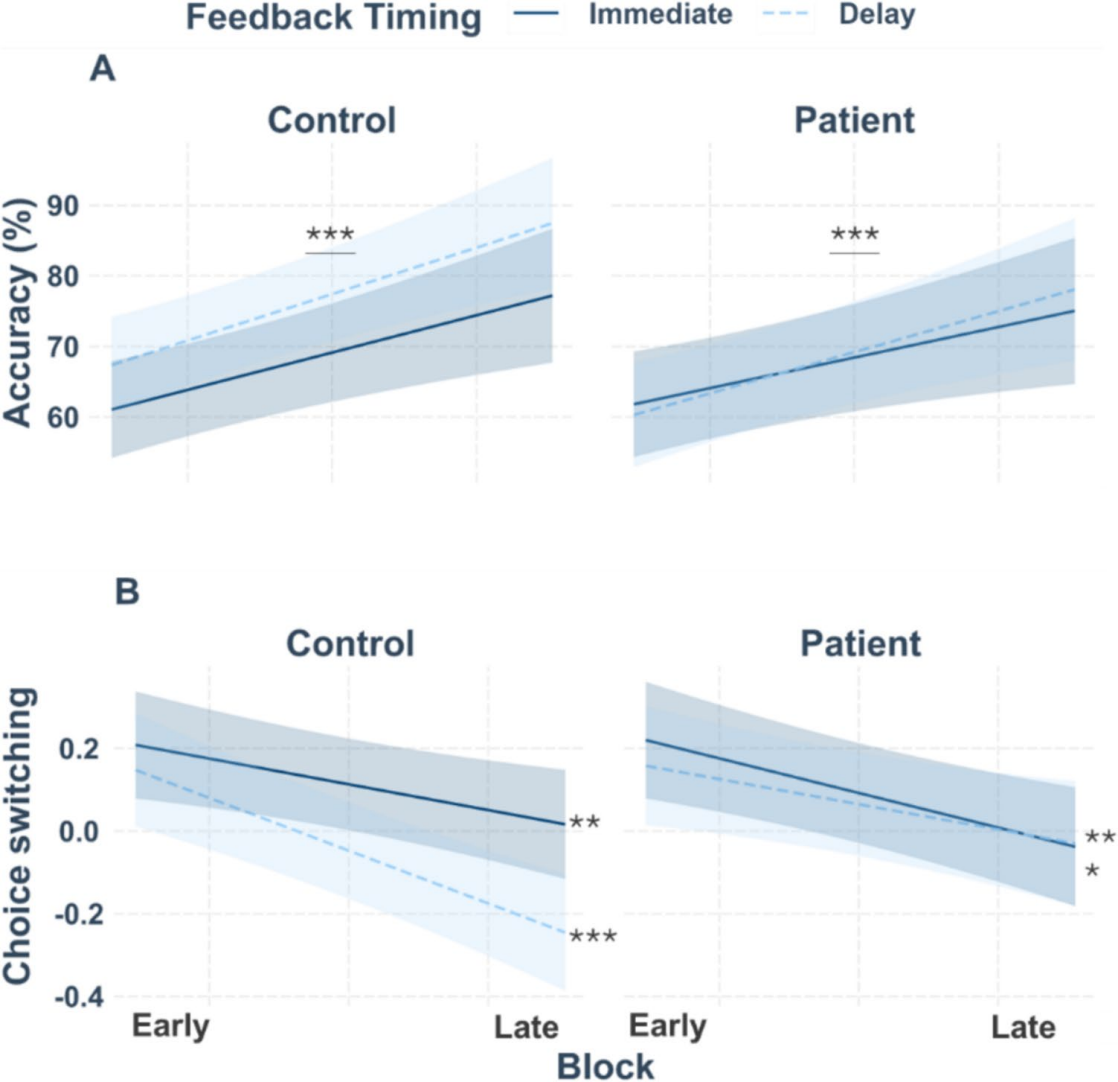
Interaction plots for accuracy (**A**) and choice switching (**B**) with the categorial factors group, feedback timing, and the scaled factor block. (**A**) For accuracy, asterisks indicate the significant main effect of block which reflects higher accuracy as the task progressed. (**B**) For choice switching, asterisks indicate significantly decreased choice switching with task progression. The strongest effect was found in controls for delayed feedback. The smoothing around the lines indicates the 95% confidence interval for *N* = 46

MLM analysis did not reveal the hypothesized difference in accuracy between patients and controls (*p* =.341). The main effect of feedback timing was significant (β = −4.52, *t*(44.00) = −2.11, *p* =.041). Across groups, accuracy was increased for delayed (*M* = 73.66 %, *SD* = 22.02 %) compared to immediate feedback (*M* = 68.81 %, *SD* = 22.10 %). The main effect of block was also significant (β = 5.50, *t*(44.00) = 7.09, *p* <.001), with lower accuracy at the beginning of the task (first block: *M* = 59.79 %, *SD* = 20.56 %) than at the end (last block: *M* = 78.38 %, *SD* = 23.05 %), indicating that learning took place. All other main and interaction effects were non-significant (all *p*-values ≥.087; *N* = 46, see Figure 2A and Table S2 in the supplement for the complete results).

### Choice switching

We also did not find the expected difference between patients and controls in choice switching (*p* =.823). However, the analysis revealed a significant main effect of feedback valence (β = −0.42, *t*(37.94) = −9.59, *p* <.001). Choice switching was reduced for positive compared to negative feedback (see Fig. 2B for the plot and Table S2 in the supplement). Likewise, choice switching was generally reduced after correct compared to incorrect choices (β = −0.34, *t*(40.37) = −4.63, *p* <.001).

The three-way interaction between feedback timing, group, and block was significant (β = −0.12, *t*(7414.71) = −1.98, *p* =.048; see Fig. 2B. Simple slopes were resolved using the factors feedback timing and group as moderators. The analysis revealed two significant block effects for both groups: decreased choice switching for immediate (controls: β = −0.06, *SE* = 0.02, *t* = −2.72,* p* =.007; patients: β = −0.08, *SE* = 0.03, *t* = −3.35,* p* <.001) and delayed feedback with task progression (controls: β = −0.13, *SE* = 0.02, *t* = −5.12,* p* <.001; patients: β = −0.06, *SE* = 0.03, *t* = −2.37,* p* =.018). In patients, the block effect (i.e., learning) was weaker for delayed feedback compared to controls. For the complete results, see the supplement (Table S3).

### Feedback-related negativity (FRN)

**Fig. 3:**
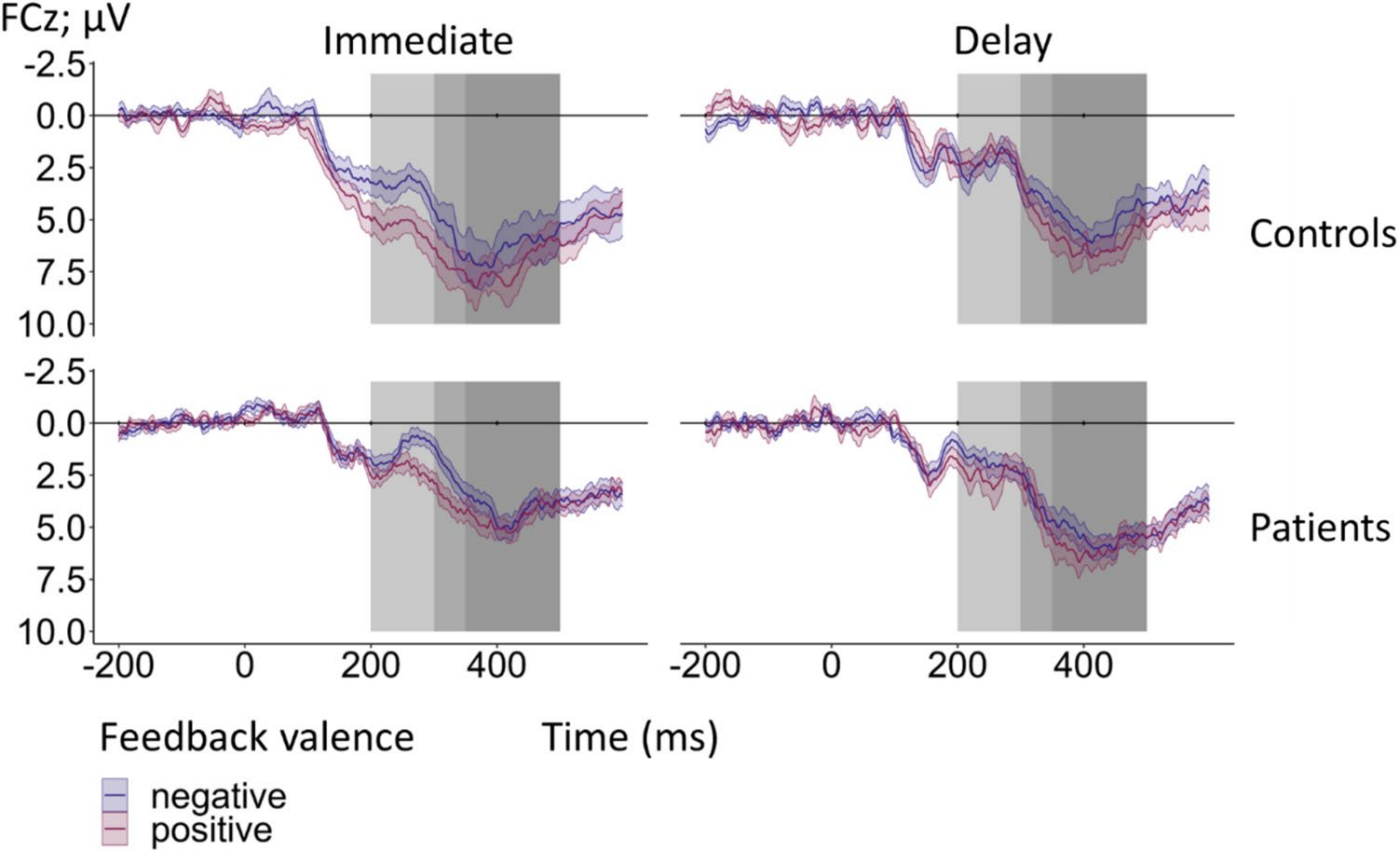
Feedback-locked grand-average ERPs at electrode FCz according to group (patients, controls), feedback timing (immediate, delayed), and feedback valence (positive, negative) for learnable trials. The light and medium gray rectangle mark the time window for the peak amplitude extraction of the FRN (200–350 ms). The P3a was quantified as mean amplitude in the time window from 300 to 500 ms as indicated by the medium and dark gray rectangle. Note that the time window from 300 and 350 ms is thus shared by FRN and P3a. Colored bands indicate standard errors. A total of 6356 trials for patients and 7206 trials for controls were averaged for the grand-averages. Detailed information on the mean number of trials included in the grand-averages according to group, feedback valence, and feedback timing are provided in the Supplement (see Table S14)

**Fig. 4:**
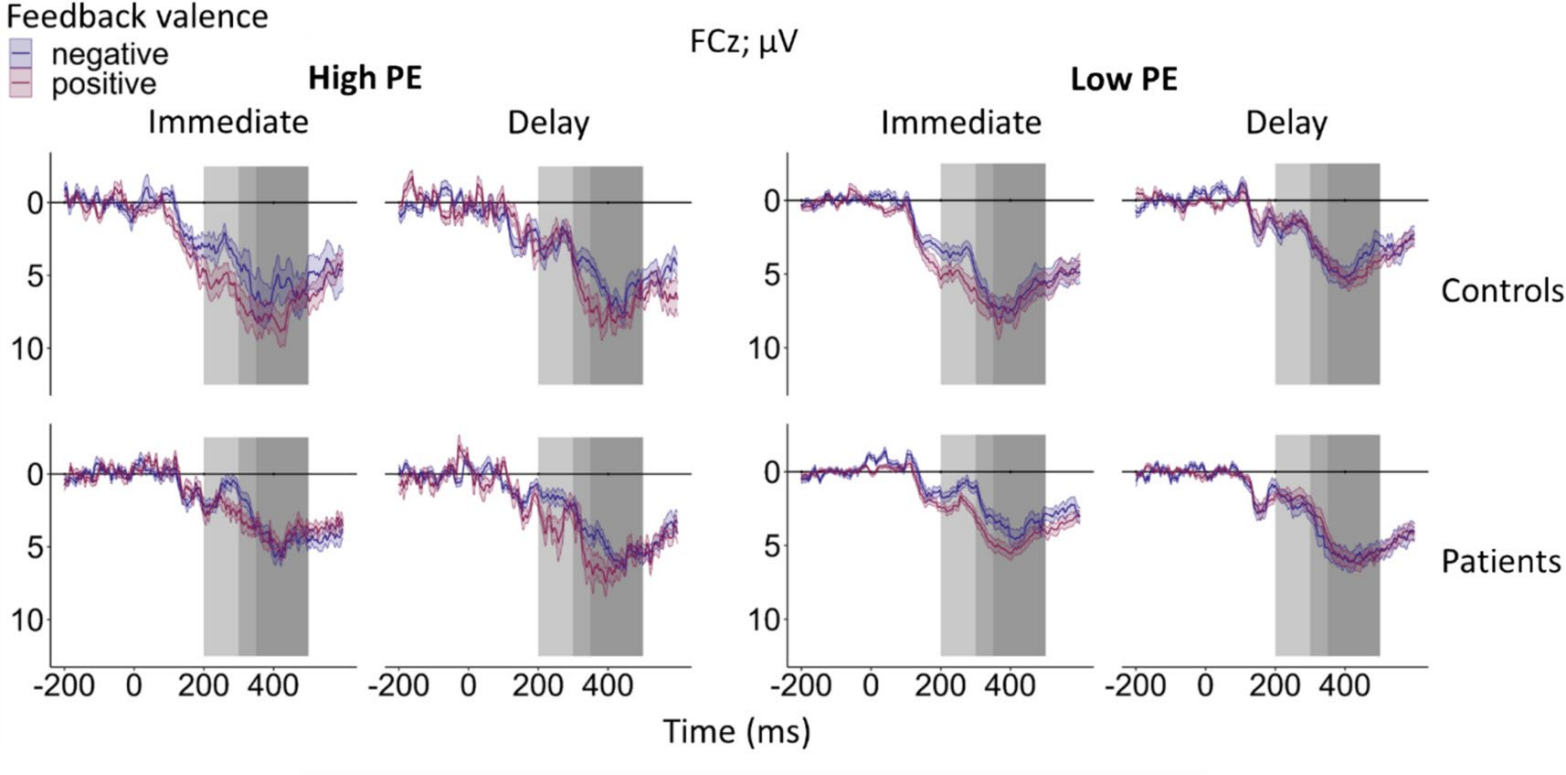
Feedback-locked grand-average ERPs for learnable trials at electrode FCz according to group (patients, controls), feedback timing (immediate, delayed), feedback valence (positive, negative), and unsigned PE, categorized into high unsigned PE (> 0.5) and low unsigned PE (≤ 0.5). The light and medium gray rectangles indicate the time window for FRN peak amplitude extraction (200–350 ms post-feedback). The P3a was quantified as the mean amplitude in the time window from 300 to 500 ms post-feedback as indicated by the medium and dark gray rectangles. Note that the time window from 300 to 350 ms post-feedback is thus shared by FRN and P3a. Colored bands indicate standard errors. Detailed information on the mean number of trials included in the grand-averages according to group, feedback valence, feedback timing, and unsigned PE can be found in the Supplement (see Table S14)

Feedback-locked grand-average ERPs at electrode FCz according to group (controls, patients), feedback timing (immediate, delayed), and feedback valence (positive, negative) for learnable trials are provided in Fig. 3. Corresponding grand-averages according to the unsigned PE (high, low) are shown in Fig. 4. Table S14 in the Supplement provides information on the mean number trials included in the grand-averages according to group, feedback timing, feedback valence, and unsigned PE.

**Fig. 5:**
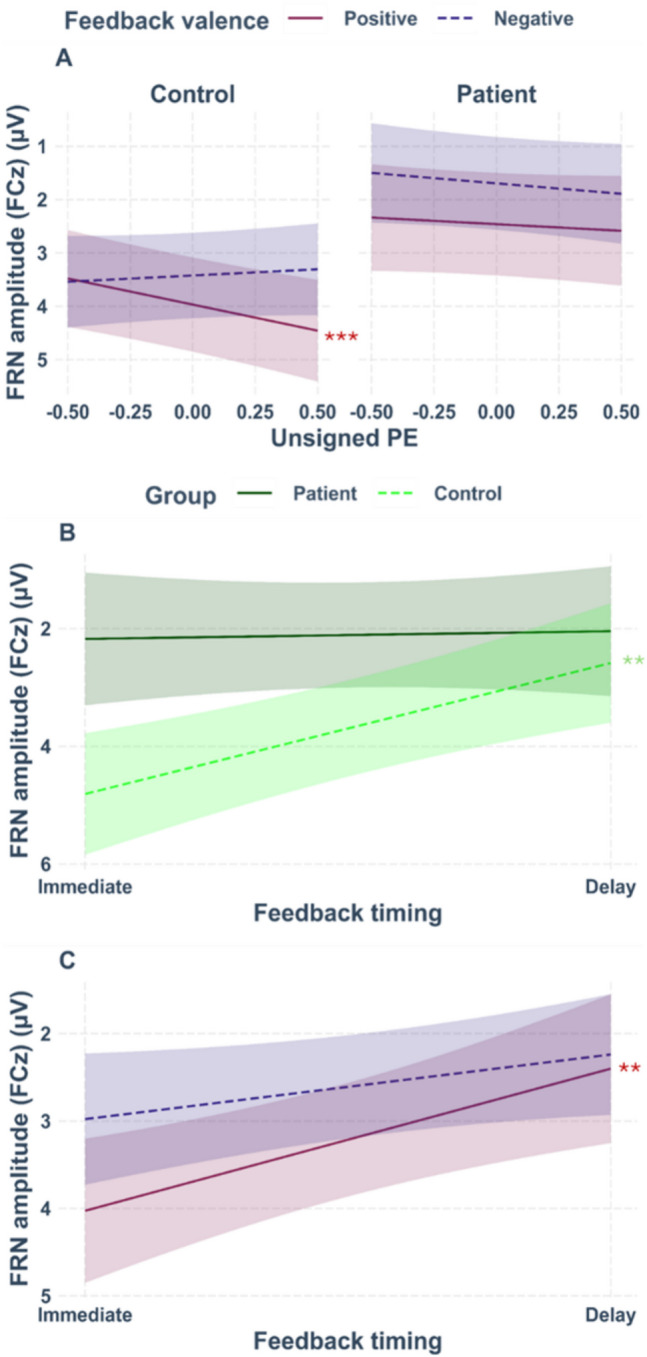
Interaction plot for single-trial FRN amplitudes at electrode FCz. **Panel A** shows the significant interaction between group, feedback valence, and unsigned PE. The slope for positive feedback modulated by the unsigned PE in controls was significant. **Panel B** shows the significant interaction between feedback timing and group. The slope for the controls modulated by feedback timing was significant. **Panel C** shows the interaction between feedback timing and feedback valence. The slope for positive feedback modulated by feedback timing was significant. Asterisks indicate significant effects. The smoothing around the lines indicates the 95% confidence interval for *N* = 46

For single-trial FRN amplitudes, altered coding of RL-PE in patients relative to controls would be reflected in a significant interaction between group, feedback valence, and unsigned PE. Indeed, a significant three-way interaction between group, feedback valence, and unsigned PE was found (β = −1.36, *t*(5672.17) = −2.04, *p* =.041, see Fig. 5A for the interaction plot). Simple slope analyses with the moderating factors group and feedback valence revealed a significant effect of the unsigned PE for controls when feedback was positive (β = 0.98, *SE* = 0.30, *t* = 3.26,* p* =.001). FRN amplitudes were more positive for higher unsigned PE. This effect was not significant for negative feedback (*p* =.461). In patients, effects of the unsigned PE were neither found for positive (*p* =.256), nor for negative feedback (*p* =.446). In addition, a main effect of group emerged (β = −1.61, *t*(44.25) = −2.64, *p* =.011), indicating a more negative FRN in patients (*M* = 2.10 µV, *SD* = 7.54 µV) compared to controls (*M* = 3.61 µV, *SD* = 8.04 µV). The interaction between group and feedback timing was also significant (β = 2.06, *t*(44.45) = 2.26, *p* =.029, see Fig. 5B for the interaction plot). Simple slope analyses with the moderating factor group revealed a significant timing effect for controls (β = −2.23, *SE* = 0.63, *t* = −3.54, *p* <.001), indicating that the FRN was more negative for delayed (*M* = 2.49 µV, *SD* = 8.25 µV) compared to immediate feedback (*M* = 4.72 µV, *SD* = 7.68 µV). For patients, the effect of feedback timing was non-significant (*p* =.848).

The FRN also showed the expected main effect of feedback valence (β = 0.65, *t*(43.61) = 3.85, *p* <.001), with more negative amplitudes for negative (*M* = 2.63 µV, *SD* = 7.75 µV) compared to positive feedback (*M* = 3.10 µV, *SD* = 7.91 µV). A full table of the statistical output can be found in Table S4 of the supplemental material.

### P3a

**Fig. 6:**
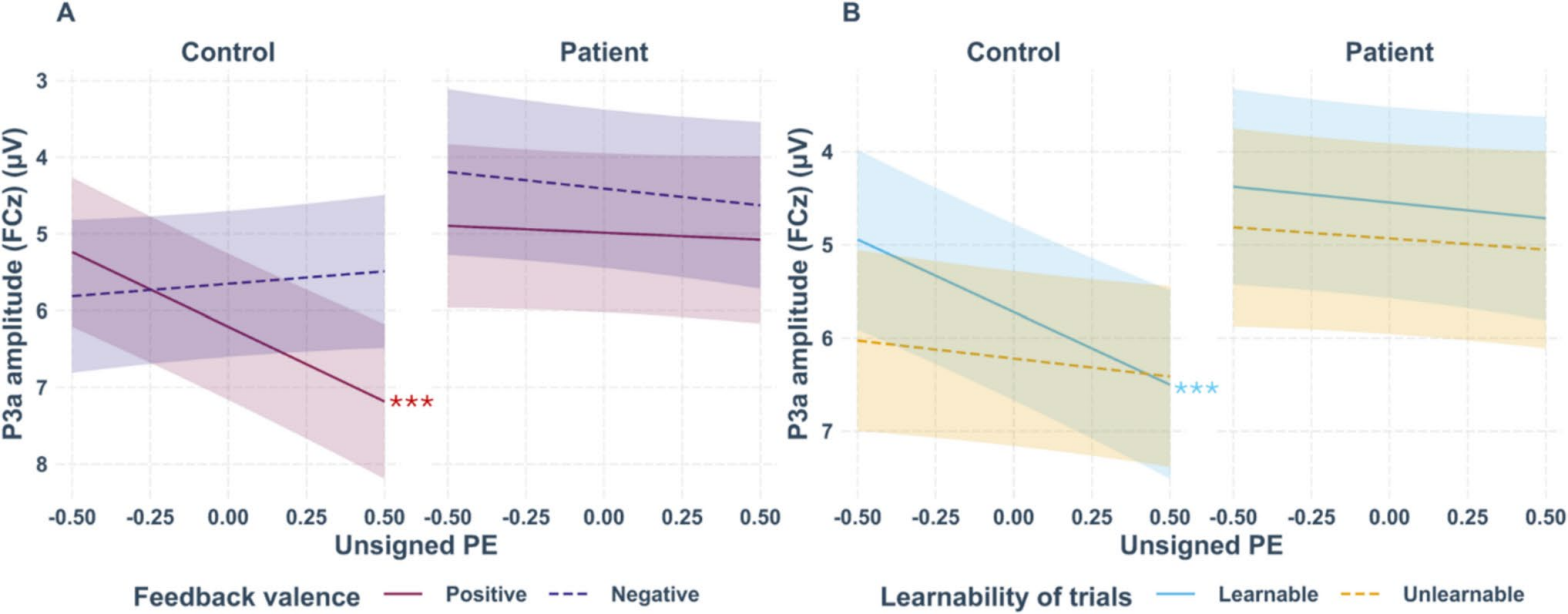
**Panel A** shows the interaction plot for single-trial P3a amplitudes at FCz according to group (patients, controls), feedback valence (positive, negative), and unsigned PE. The PE effect for positive feedback modulated by group and unsigned PE was significant in the control group. **Panel B** shows the significant interaction between group, learnability (learnable, unlearnable), and unsigned PE. The effect on learnability modulated by group and unsigned PE was significant in controls. Asterisks indicate significant effects. The smoothing around the lines indicates the 95% confidence interval for *N* = 46

Similar to the FRN, we discovered a significant three-way interaction between group, feedback valence, and unsigned PE (β = −2.53, *t*(26811.17) = −3.95, *p* <.001, see Figs. 3 and 4 for the grand-averages separately for high and low PE, and Fig. 6A for the interaction plot). Simple slope analysis with the moderating factor group revealed a significant effect for positive feedback only for controls (β = 1.95, *SE* = 0.28, *t* = 7.01, *p* <.001), with more positive P3a amplitudes for higher unsigned PEs. All other simple slopes were non-significant (all *p*-values ≥.196). A significant three-way interaction was also present between the factors group, learnability, and unsigned PE (β = −1.18, *t*(18997.71) = −2.10, *p* =.036, see Fig. 6B for the interaction plot). Simple slope analysis with the moderating factor group revealed again a significant effect for learnable trials only in controls (β = 1.56, *SE* = 0.29, *t* = 5.28, *p* <.001), with more positive P3a amplitudes for higher unsigned PEs. All other simple slopes were non-significant (all *p*-values ≥.124). A significant main effect of feedback valence was present (β = 0.57, *t*(45.88) = 4.03, *p* <.001). The P3a was increased for positive (*M* = 5.41 µV, *SD* = 7.86 µV) compared to negative feedback (*M* = 5.03 µV, *SD* = 8.10 µV). Table S5 in the supplemental material contains the complete statistical results.

### P3b

**Fig. 7:**
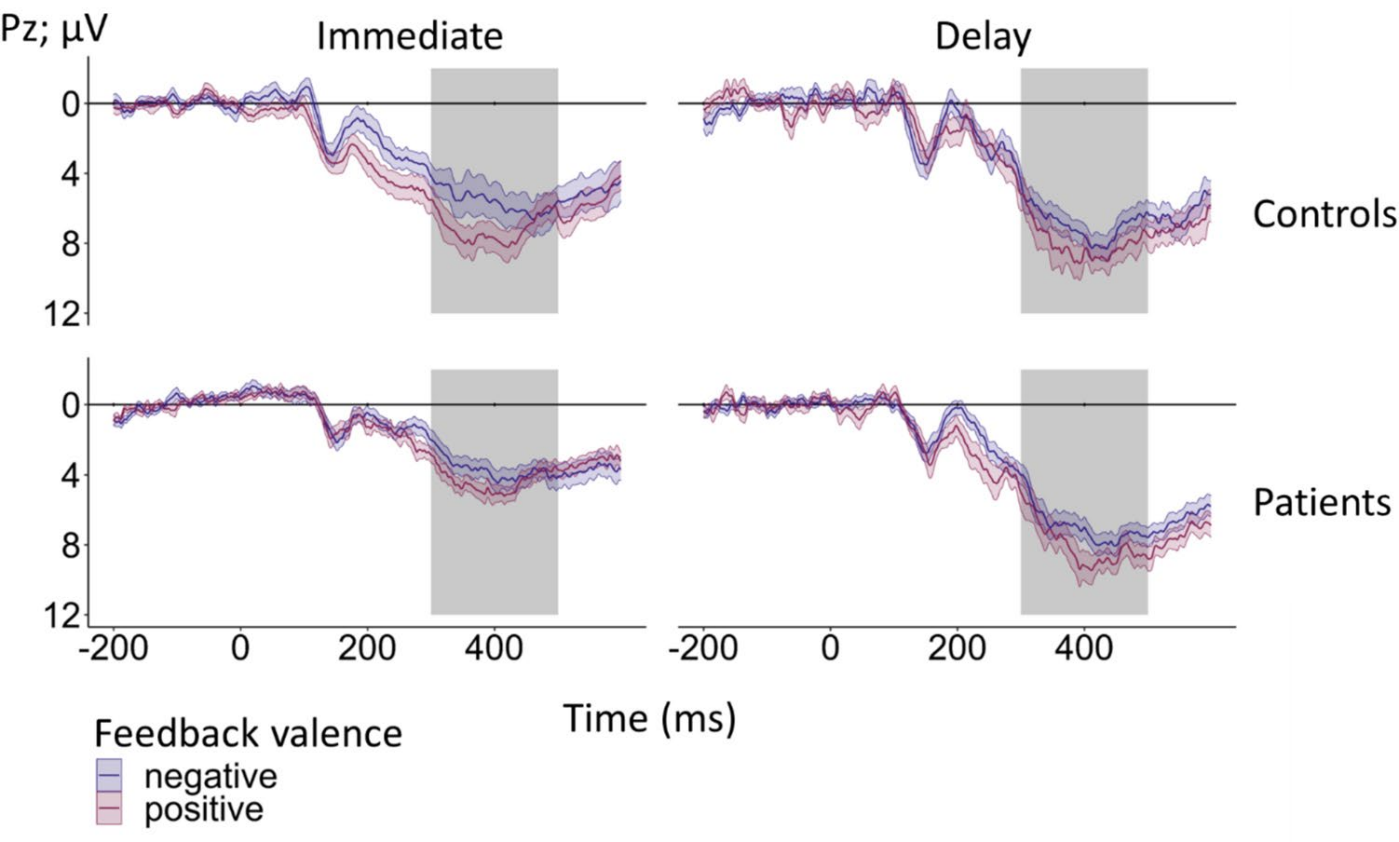
Feedback-locked grand-average ERPs for learnable trials at electrode Pz according to group (patients, controls), feedback timing (immediate, delayed), and feedback valence (positive, negative). The gray rectangle indicates the time window for P3b mean amplitude quantification (300 to 500 ms post-feedback). Colored bands indicate standard errors. Detailed information on the mean number of trials included in the grand-average according to group, feedback valence, feedback timing, and unsigned PE is provided in the Supplement (see Table S14)

**Fig. 8:**
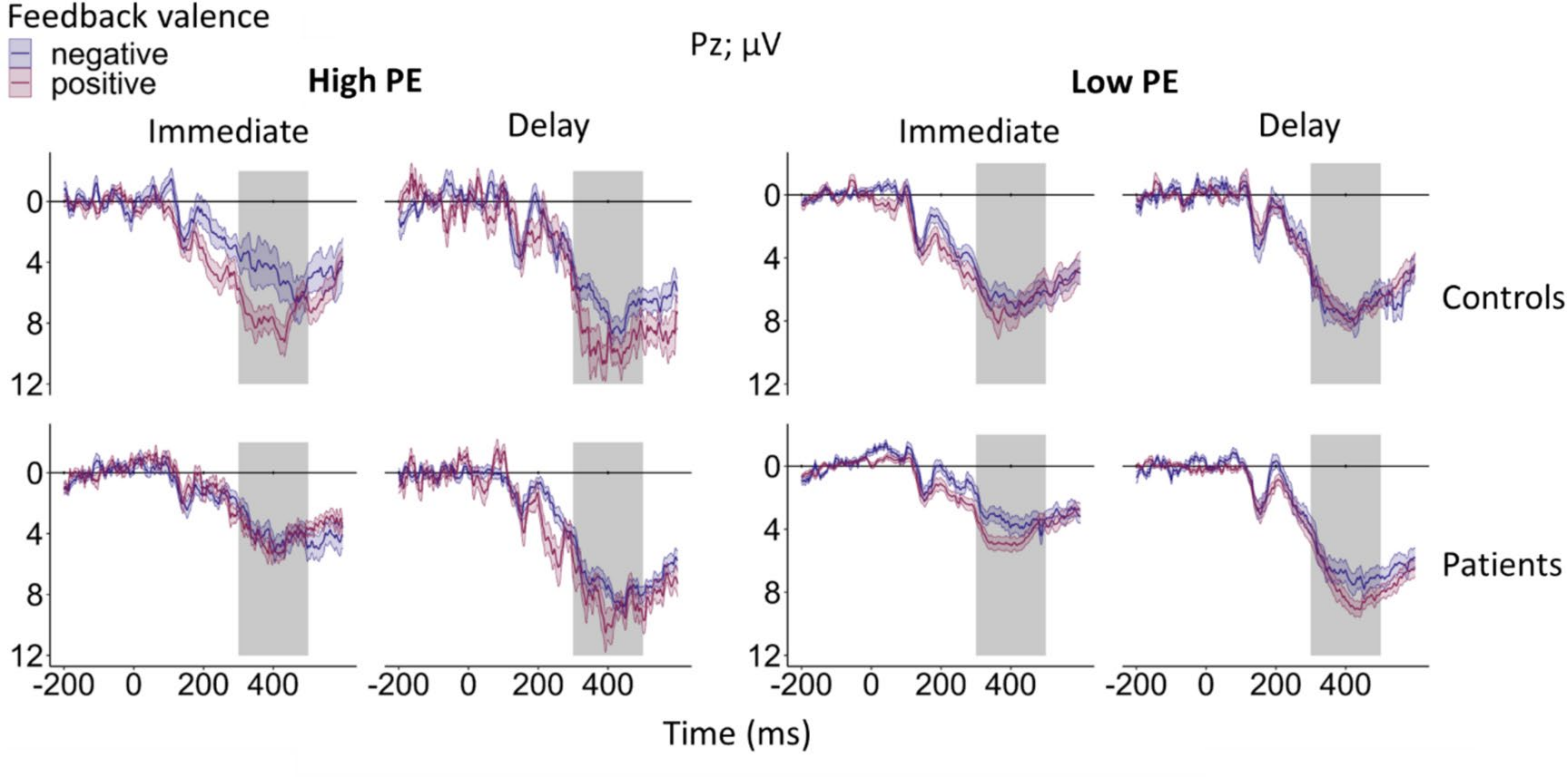
Feedback-locked grand-average ERPs for learnable trials at electrode Pz according to group (patients, controls), feedback timing (immediate, delayed), feedback valence (positive, negative), and unsigned PE categorized into high unsigned PE (> 0.5) and low unsigned PE (≤ 0.5). The gray rectangle indicates the time window for P3b mean amplitude quantification (300 to 500 ms post-feedback). Colored bands indicate standard errors. Detailed information on the mean number of trials according to group, feedback valence, feedback timing, and unsigned PE can be found in the supplement (see Table S14)

Feedback-locked grand-average ERPs for learnable trials at electrode Pz according to group (controls, patients), feedback timing (immediate, delayed), and feedback valence (positive, negative) are provided in Fig. 7. Corresponding grand-averages according to the unsigned PE (high, low) are depicted in Fig. 8.

**Fig. 9:**
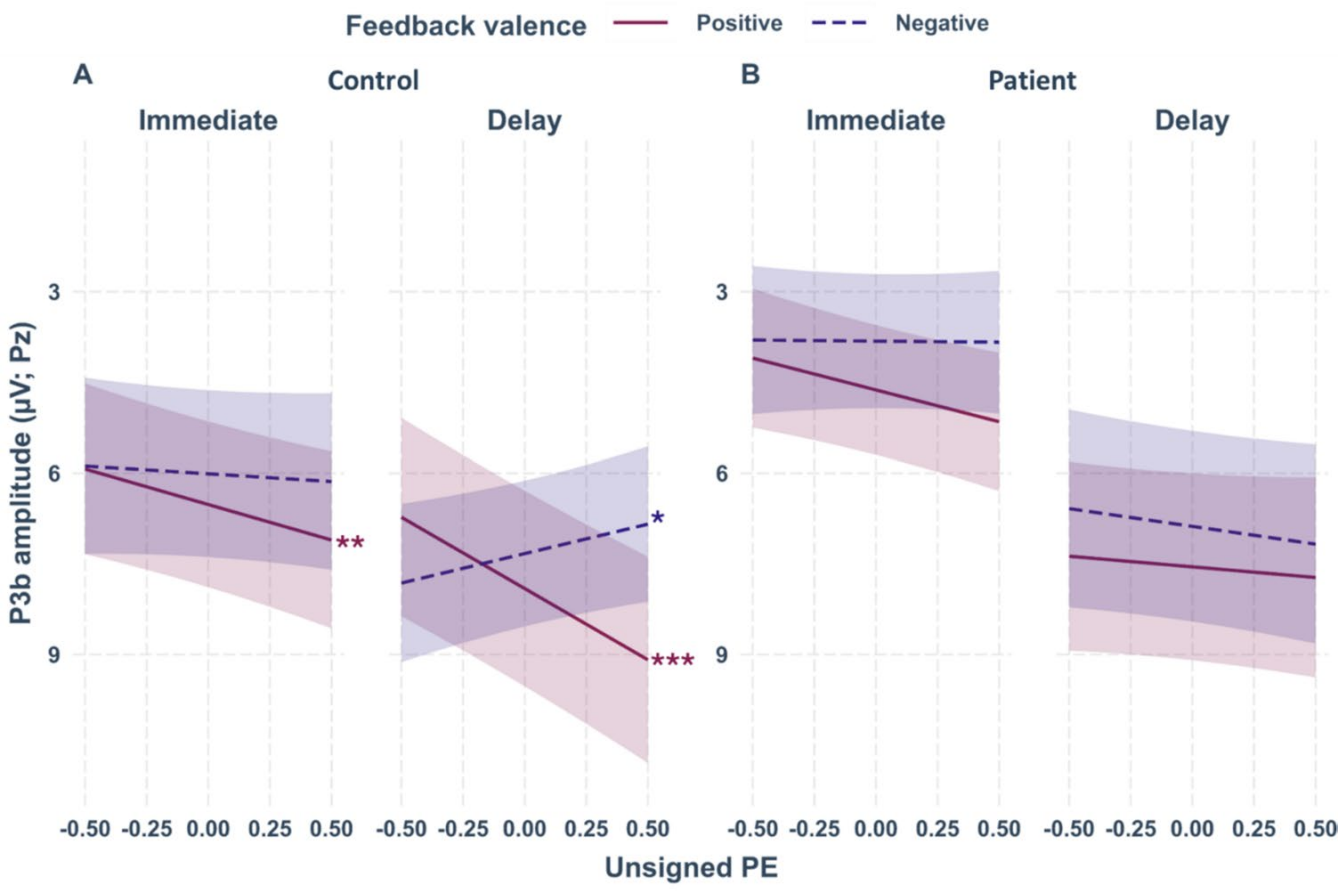
Interaction plots for the single-trial P3b at electrode Pz. The categorical factors are feedback timing (immediate, delay), feedback valence (positive, negative), and the continuous factor is unsigned PE. **Panel A**: the effects for positive immediate and positive and negative delayed feedback in the control group were significant. Asterisks indicate significant effects. The smoothing around the lines indicates the 95% confidence interval for *n* = 25. **Panel B** shows the plot of the non-significant three-way interaction in patients (*n* = 21). The significance of the slopes is therefore not highlighted or further interpreted

For the single-trial P3b, we found a significant four-way interaction between group, feedback timing, feedback valence, and unsigned PE (β = −3.79, *t*(23933.17) = −2.71, *p* =.007). To resolve this complex interaction, we created separate models for patients and controls. For patients, the three-way interaction between feedback timing, feedback valence, and unsigned PE was non-significant (*p* =.170). For controls, the three-way interaction between feedback timing, feedback valence, and unsigned PE was significant (β = 2.42, *t*(13684.19) = 2.55, *p* =.011, see Fig. 9 for the interaction plot). Simple slope analysis moderated by feedback timing and feedback valence revealed a significant effect of the unsigned PE for positive, delayed feedback (β = 2.36, *SE* = 0.44, *t* = 5.43, *p* <.001). P3b amplitudes were more positive, i.e. increased, for higher unsigned PEs when feedback was positive and delayed. In addition, a significant negative effect of the unsigned PE for negative, delayed feedback was present (β = −0.97, *SE* = 0.49, *t* = −2.01, *p* =.045). For negative, delayed feedback, P3b amplitudes decreased with higher unsigned PEs. A positive effect of the unsigned PE for positive, immediate feedback was also significant (β = 1.18, *SE* = 0.44, *t* = 2.65, *p* =.008), indicating more positive P3b amplitudes for higher unsigned PEs. The effect for negative, immediate feedback was non-significant (*p* =.595). The full results tables for the main model on the P3b (see Table S6) and subordinate group-specific models (see Table S7 for patients and S8 for the controls) are provided in the supplemental material.

### Voxel-based morphometry (VBM)

**Fig. 10:**
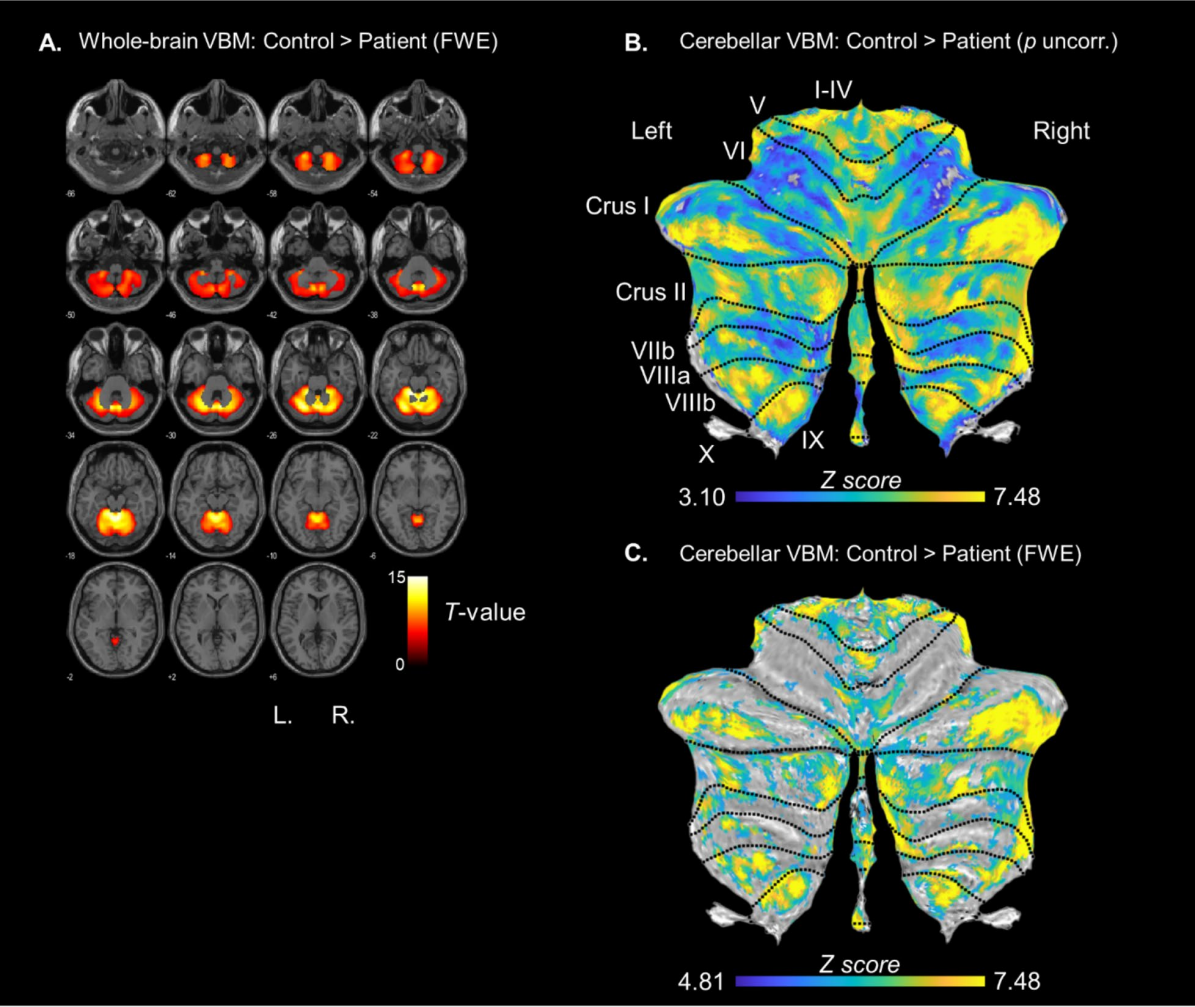
**Panel A**: Whole-brain GMV reduction in patients compared to controls. **Panel B**: Cerebellar GMV reduction in patients relative to controls (SUIT space) uncorrected (*p* <.001) and in **Panel C** after FWE-correction (for* p* < 0.05) projected on the cerebellar flatmap (Diedrichsen & Zotow, 2015). TIV and age were used as covariates of no interest. The color bars indicate the range of *T*-values for whole brain and z scores for the cerebellar flatmaps

The analysis of GMV in patients (*n* = 18) and controls (*n* = 24) revealed the expected significant volume reduction in patients in widely distributed cerebellar clusters (see Fig. 10A and Table S10 and S11 in the supplement for the whole-brain results, Figure 10B for the cerebellar results uncorrected, Fig. 10C for the FWE-corrected results; Table 3 provides peak coordinates of the largest cluster after FWE correction in SUIT-space). Note that there were no extracerebellar clusters with significant volume reduction in patients relative to controls. Cerebellar VBM revealed the most pronounced GMV reduction in posterolateral regions of the cerebellum (here shown for cluster size > 500 voxels) in right Crus I (1452 voxels), right Crus II (1401 voxels), left Crus II (872 voxels), right I-IV (828 voxels), right IX (742 voxels), left I-IV (706 voxels), left Crus I (677 voxels), and left IX (563 voxels, see Table S9 in the supplement for a complete list of clusters and Table S10 for the list of uncorrected clusters).

**Table 3:** Summary of the six local maxima for the largest cluster of the between-subjects contrast controls > patients

location	side	X	Y	Z	peak *p*-value	peak *t*-value
VI	right	29	−38	−35	*p* <.001	11.43
VIIb	right	35	−43	−44	*p* <.001	10.13
Crus I	left	−39	−67	−36	*p* <.001	10.00
Crus II	left	−8	−80	−39	*p* <.001	9.80
I-IV	right	11	−44	−25	*p* <.001	9.52
IX	right	10	−50	−46	*p* <.001	9.46

*Note*. Covariates of no interest were TIV and age. The cluster size was 11869 voxels. Results were FWE-corrected for *p* < 0.05. A complete list of significant regions can be found in the supplement Table S9

**Fig. 11:**
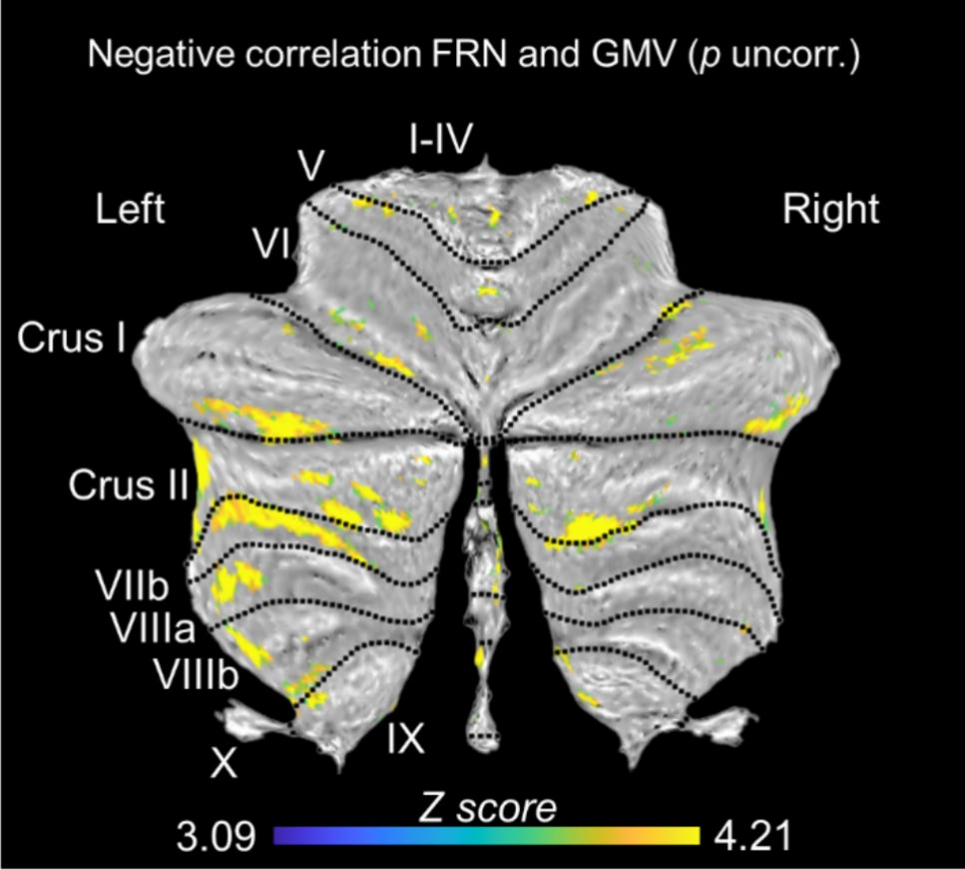
Clusters in which cerebellar GMV loss in patients relative to controls was linked to blunting of the FRN (aggregated across all single trials). The biggest cluster was present in left Crus II. TIV and age were used as covariates of no interest. The color bar indicates the range of z-scores. All identified clusters were uncorrected *p* <.001

Multiple regression analysis revealed that volume reduction in bilateral Crus I (left = 266, right = 103 voxels) and Crus II (left = 620, right = 249 voxels) was associated with more positive (i.e., blunted) FRN amplitudes (here shown for cluster size > 100 voxels, see Fig. 11 and Table S12 for all clusters in the supplement).

## Discussion

The goal of the present study was to investigate feedback-based learning and RL-PE processing in patients with pure cerebellar degeneration, with a focus on potential effects of feedback timing. To this end, EEG was recorded while participants completed a probabilistic feedback-based learning task in two sessions with different feedback timings, i.e., immediate feedback (delay = 500 ms) or delayed feedback (delay = 6500 ms). FRN, P3a, and P3b in the feedback-locked ERP were analyzed in relation to (modelled) unsigned PEs for each individual trial. VBM was conducted on whole-brain data and in a separate analysis for the cerebellum to characterize GMV volume reduction in patients relative to controls, and to potentially link specific cerebellar regions to group differences in task performance and/or EEG measures reflecting RL-PE processing.

Analysis of the behavioral data revealed that accuracy increased with task progression, indicating that learning took place gradually. Importantly, we did not find the hypothesized group differences, nor differential effects of feedback timing. Given that accuracy was generally increased for delayed feedback, this finding may hint at decreased uncertainty when feedback was delayed. We had hypothesized that the cerebellum may be differentially involved in learning from feedback as a function of timing. The present results do not support this notion. Of note, a recent study in patients with cerebellar stroke reported similar results (Huvermann et al., 2025). The present study found only subtly reduced behavioral flexibility in patients as reflected in reduced choice switching: While in controls, decreased choice switching for delayed relative to immediate feedback was found in later task stages, such an effect was absent in patients. This pattern might indicate that choice switching was not modulated by the learning progress in patients, possibly due to decreased behavioral flexibility as has been reported in patients with cerebellar damage for rule- and reversal learning tasks (see Thoma et al., 2008; for a review see Berlijn, Huvermann, Schneider et al., 2024b).

EEG analyses revealed a typical modulation of the FRN by feedback valence, with increased negativity for negative relative to positive feedback (e.g., Gehring & Willoughby, 2002; Nieuwenhuis et al., 2005; Pfabigan et al., 2011). In addition, in line with the expectations, the unsigned PE was reflected in the FRN, with more negative amplitudes for more unexpected feedback (i.e., higher unsigned PE; e.g., Chase et al., 2011; Walentowska et al., 2019). In addition, the FRN was more negative for delayed compared to immediate feedback which is in line with previous results (Arbel et al., 2017; Peterburs et al., 2016; Weismüller & Bellebaum, 2016; Weber & Bellebaum, 2024).

As hypothesized, the unsigned PE was not reflected in the FRN in patients, while it was for controls, albeit only for positive feedback. The pattern in controls is consistent with recent reports of modulation of the FRN by positive PEs (Kirsch et al., 2022; Weber & Bellebaum, 2024), indicating that coding of high PEs (i.e., unexpected feedback) in the FRN was more pronounced for positive compared to negative feedback. This is consistent with the view that the signal in the FRN time window is driven by a RewP (Weber & Bellebaum, 2024). In this regard, one advantage of the present study was using feedback valence and the unsigned PE as separate factors, allowing us to investigate PE effects in patients and controls as a function of feedback valence.

Lack of PE coding in the FRN in patients (in both timing conditions) could be indicative of a general deficit in RL-PE processing. In line with this notion, patients did show an overall increased FRN that could hint at increased unexpectedness independent of the PE during the task. Evidence for impaired coding of surprise, which can also be interpreted as deficit in RL-PE processing, has previously been found in cerebellar stroke (Rustemeier et al., 2016). However, in this study, PEs were not modelled at single-trial level. In contrast, Huvermann et al. (2025) did model RL-PEs and found RL-PE coding to be absent in cerebellar stroke patients compared to controls. Further supporting the notion that cerebellar dysfunction impairs RL-PE processing, RL-PE coding was also lacking when healthy subjects received cerebellar TMS (Huvermann et al., 2025). Together with the present findings, these results evidence cerebellar involvement in processing of RL-PEs as indexed by the FRN. Of note, this conclusion is further supported by a recent meta-analysis on PE processes in humans that discovered an association between unsigned PEs and cerebellar activation, among other regions (Corlett et al., 2022). For the signed PE, cerebellar effects were not found.

The FRN was not the only ERP component sensitive to RL-PE processing in the present study. Analysis of the P3a revealed similar result patterns, with increased positivity with higher unsigned PE in controls for positive feedback. This finding is in line with recent studies in which the frontal P300/P3a reflected the PE for positive immediate feedback (Kirsch et al., 2022; Weber & Bellebaum, 2024). It is also consistent with results reported by Hoy et al. (2021). Of note, these authors not only found the P3 to represent an unsigned PE in healthy subjects (Hoy et al., 2021), they also observed a more central scalp distribution (consistent with the P3a) when analyzing the magnitude of the RL-PE solely by positive feedback. Functionally, the P3a has been linked to attentional reorienting and has been suggested to encode expectancy (Chase et al., 2011; Walentowska et al., 2019). Along these lines, PE effects in the P3a in the present study could be interpreted as a surprise response in controls for immediate positive feedback that was absent in patients. Importantly, the present effects of the P3a could be also influenced by the RewP because the time windows of both ERP components overlap to some extent (Ullsperger, 2024).

We did not find an effect of feedback timing for the P3a, which is in accordance with findings by Höltje and Mecklinger (2020). In contrast, the later P3b was sensitive to feedback timing, albeit as a function of feedback valence, the unsigned PE, and group. This is particularly relevant because functionally the P3b has been implicated in updating of context-related information (Polich, 2007), and also in PE processing directly (Lauffs et al., 2020). Stewardson and Sambrook (2020) calculated great grand-averages across multiple studies and found that a parietal scalp deflection related to reward PE processing was stronger than an earlier frontal effect, underlining the significance of the P3b for PE processing. In line with this, the unsigned PE was reflected in the P3b in controls in the present study, with increased positivity for higher PEs, particularly for positive, immediate feedback. Moreover, differential patterns emerged for positive and negative delayed feedback: P3b amplitudes were more positive for higher positive PEs but decreased with higher negative PEs. Crucially, coding of the unsigned PE in the P3b was completely absent in patients. Together with largely absent PE coding in patients also in FRN and P3a, this result appears to indicate a rather global alteration of feedback-related ERPs in cerebellar degeneration regarding RL-PE processing. Importantly, other aspects of feedback processing such as valence coding were intact, arguing against a global alteration of the ERP in patients per se.

Interestingly, patients did show a generally more positive P3b for delayed feedback, which might be driven by the functional role of the P3b for updating contextual information. Höltje and Mecklinger (2020) found a more pronounced P3b for immediate compared to delayed feedback in healthy subjects and linked this to increased action value updating when feedback was presented immediately. Healthy subjects in the present study did not show an effect of feedback timing, but we observed an effect in patients, with a decreased P3b for immediate compared to delayed feedback. It is conceivable that context updating is more demanding for longer delay duration due to higher working memory demand. Working memory impairment is a common non-motor symptoms in patients with cerebellar lesions (Hoche et al., 2018; Peterburs et al., 2010). An fMRI study that used an n-back task in patients with cerebellar lesions and healthy controls suggested that increased, likely compensatory, activations in parietal areas in patients may underly preserved task performance (Ziemus et al., 2007).

As discussed so far, the present results suggest altered neural responses, particularly with regard to RL-PE processing, in patients with cerebellar degeneration, which, however, are only accompanied by subtle behavioral impairment. As expected, whole-brain VBM results showed significant GMV reduction in patients compared to controls spanning wide regions of the cerebellum. Importantly, there were no (structural) extra-cerebellar differences between patients and controls. Cerebellar VBM using SUIT showed the strongest GMV reduction in bilateral Crus I/II and other posterolateral regions of the cerebellum. This is important because particularly Crus I and II have been linked to cognitive functions (Stoodley, 2012; Stoodley & Schmahmann, 2010). According to the functional atlas by King et al. (2019) and van Overwalle et al. (2023), Crus II is particularly implicated in action observation and understanding. In the present study, GMV reduction particularly in bilateral Crus I/II was associated with more positive FRN amplitudes. At first glance, this finding appears surprising, given generally more negative FRN amplitudes in patients compared to controls. However, blunting of the FRN (i.e., decreased negativity) with increasing GMV reduction is consistent with previous observations for the response-locked ERP component ERN in patients with cerebellar degeneration (Peterburs et al., 2015). It also conforms to recent findings in healthy subjects in whom cerebellar function was disrupted by single-pulse TMS applied to the posterolateral cerebellum (Berlijn, Huvermann, Groiss et al., 2024a). Both, the FRN and ERN originate from the ACC (Hauser et al., 2014; Herrmann et al., 2004) and are closely linked to RL-PE processing (Holroyd & Coles, 2002).

In general, the dissociation between preserved behavior and altered neural responses warrants further discussion (Ullsperger, 2024). Kirsch et al. (2022), contrary to their hypotheses, only found a small association between behavior and the FRN and argued that this could be due to the information content of feedback in their task design. Walsh and Anderson (2012) also discussed in their review the absence of behavioral findings across different studies while effects on the FRN were demonstrated. They hypothesized that the FRN could reflect a habitual behavioral response rather than goal-directed behavior. Given these different perspectives on the nature of the FRN and the potential separation into an N200 and the RewP as independent components that are differently affected by expectancy and feedback valence (Ullsperger, 2024), the exact role of the FRN and therefore its link to behavior is not yet fully understood (Kirsch et al., 2022). It must be noted that the present task used reward probabilities that were constant throughout the task, and coding of feedback valence was intact in patients, so small changes in PE might not have been necessary for learning (unlike in reversal learning tasks). In contrast to the brain-behavior dissociation in the FRN, P3a and P3b have been linked to behavioral adaptation by Kirsch et al., (2022). Here, the frontal P3a appeared to be less positive when more behavioral adaptation for the next trial was necessary. Also, unexpected feedback led to increased P3b amplitudes, suggesting a role of the P3b for updating of action values. In contrast, the present study did not reveal a relation between absent coding of RL-PEs in P3a and P3b and behavioral performance in the patient sample in comparison to controls.

### Limitations

The present study was designed to characterize the cerebellum’s role in reinforcement learning and coding of RL-PEs as a function of feedback timing by investigating patients with different ataxia disorders characterized by progressive cerebellar degeneration. Including patients with etiologically different diseases might have led to increased (unexplained) variance in our results that was particularly problematic for the VBM. However, we used a homogeneity analysis to exclude participants with extreme cerebellar GMV reduction to cope with strong variance differences. It can be discussed whether the included neurodegenerative diseases described as purely cerebellar also affect other regions of the brain, as has been recently reported for patients with SCA6 who showed an increased concentration of iron in the basal ganglia which correlated with lower cognitive performance (Marvel et al., 2022). Also, the discrepancy between the group effect in the FRN for the MLM and the negative correlation in the VBM could have been the result of the aggregated data points for the FRN in the GLM. While MLMs model each individual trial for each participant, the GLM models only the aggregated values of the FRN for each participant. Hence, individual variability is lost with the GLM. In addition, we did not see the expected differences in CCAS scores between patients and controls (see Table 2). This is in line with previous findings by Thieme et al. (2022) who showed that although SCA6 patients scored numerically lower than controls, the group difference was not significant (Thieme et al., 2022). Both the CCAS scale and our task might not have been sensitive enough to find behavioral differences. General learning performance in the present study was comparable to previous studies, with accuracy in the acquisition stage ranging between 50 % and 80 % in Thoma et al. (2008) and Rustemeier et al. (2016). Of note, we did not include reversal learning which was shown to be altered in patients with cerebellar lesions (Thoma et al., 2008). Thus, our task design with constant reward probabilities unfortunately does not allow for insights in this direction beyond the behavioral findings on trial-by trial choice switching. Last, the time windows for FRN and P3a extraction overlapped by 50 ms which could contribute to diminished functional distinction if feedback processing is assumed to be initially reflected in the FRN and later in the P3a (Ullsperger, 2024). Including FRN and P3a as separate measures is in line with many previous studies on feedback-based learning (e.g., Mangels et al., 2018; Tilton-Bolowski et al., 2021). In the present study, these two components were scored differently. The FRN was scored as mean amplitude in a time window of ±20 ms around the FRN peak latency that was determined in individual averages. The P3a was scored as mean amplitude in the time window from 300 to 500 ms post-feedback. Importantly, the average FRN peak latency was 257.29 ms (*SD* = 30.95 ms) for the entire sample, thus clearly preceding the P3a time window so that FRN results should not have been a confound for P3a results.

## Conclusion

In conclusion, the present results revealed altered RL-PE processing in probabilistic feedback-based learning in patients with pure cerebellar degeneration. Analyses of FRN, P3a, and P3b in the feedback-locked ERP revealed absent coding of RL-PEs in patients. Whole-brain and cerebellar VBM showed global cerebellar degeneration in patients compared to controls, and multiple regression revealed that reduced GMV in bilateral Crus I/II was associated with blunting of the FRN. Importantly, the present results did not provide evidence for differential involvement of the cerebellum in reinforcement learning or feedback processing as a function of feedback timing. Nevertheless, the present results underline the cerebellum’s role in RL-PE processing. More research is needed to fully elucidate the mechanisms of cerebellar contributions to PE processing as well as contextual factors that may modulate these processes using task-based fMRI, particularly to disentangle the (cerebro-cerebellar) networks underlying reinforcement learning in healthy and diseased cerebellum.

## Supplementary Information

Below is the link to the electronic supplementary material.

Supplementary file1 (DOCX 446 KB)

## Data Availability

The study protocol was defined prior to the experiment and preregistered on OSF. The preregistration, data, and code are openly available through OSF at https://osf.io/fgw8h/
