# Supplementary material for: Impaired reinforcement learning and coding of prediction errors in patients with cerebellar degeneration - a study with EEG and voxel-based morphometry
Source: Cogn Affect Behav Neurosci. 2025 May 28;25(4):1126–46. doi: 10.3758/s13415-025-01303-2 (PMC12356735; doi:10.3758/s13415-025-01303-2)
Supplement: Supplementary file 1 — Supplementary file1 (DOCX 446 KB) [file 13415_2025_1303_MOESM1_ESM.docx]

**Supplemental material:**

**Impaired reinforcement learning and coding of prediction errors in patients with cerebellar degeneration - a study with EEG and voxel-based morphometry**

Adam M. Berlijn^1,2,3^, Dana M. Huvermann^1,4^, Eric Bechler^5^, Andreas Thieme^4^, Alfons Schnitzler^2,6^, Christian Bellebaum^1^, Dagmar Timmann^4^, Martina Minnerop^2,3,6^, and Jutta Peterburs^1,7^

^1^Faculty of Mathematics and Natural Sciences, Heinrich Heine University Düsseldorf, Düsseldorf, Germany

^2^Institute of Clinical Neuroscience and Medical Psychology, Medical Faculty & University Hospital Düsseldorf, Heinrich Heine University Düsseldorf, Düsseldorf, Germany

^3^Institute of Neuroscience and Medicine (INM-1), Research Centre Jülich, Jülich, Germany

^4^Department of Neurology and Center for Translational and Behavioral Neurosciences (C-TNBS), Essen University Hospital, University of Duisburg-Essen, Essen, Germany

^5^Core Facility for Magnetic Resonance Imaging, Medical Faculty and University Hospital Düsseldorf, Heinrich Heine University Düsseldorf, Düsseldorf, Germany

^6^Department of Neurology, Center for Movement Disorders and Neuromodulation, Medical Faculty & University Hospital Düsseldorf, Heinrich Heine University Düsseldorf, Düsseldorf, Germany

^7^Institute of Systems Medicine and Department of Human Medicine, MSH Medical School Hamburg, Hamburg, Germany

^1^Corresponding author: [berlijn@uni-duesseldorf.de](mailto:berlijn@uni-duesseldorf.de), +49 211 81-14799

Department of Experimental Psychology

Heinrich Heine University Düsseldorf

Universitätsstraße 1, 40225

Düsseldorf, Germany

**Table of Contents**

| Content | page |
| --- | --- |
| **Power analysis** | **3** |
| **Neurological and neuropsychological assessment** | **3** |
| Results of the neurological and neuropsychological assessment |  |
| Table S1 |  |
| **Complete inferential statistics for the MLMs reported in the main manuscript** | **5** |
| **Accuracy** | 5 |
| Table S2 |  |
| **Choice switching** | 6 |
| Table S3 |  |
| **FRN** | 8 |
| Table S4 |  |
| **P3a** | 10 |
| Table S5 |  |
| **P3b** | 12 |
| Table S6, S7, S8 |  |
| **Homogeneity check in the patient sample** | **16** |
| Boxplot of the homogeneity analysis of the patient sample | 16 |
| Figure S1 |  |
| Visual inspection of the GMV in SUIT-space for the patient sample. | 17 |
| Figure S2 |  |
| **VBM analyses: Complete cluster lists** | **18** |
| Whole-brain - Between group analysis | 18 |
| Table S9 |  |
| SUIT – Between group analysis FWE-corrected | 19 |
| Table S10 |  |
| SUIT – Between group analysis *p*-uncorrected | 20 |
| Table S11 |  |
| SUIT – Multiple regression FRN | 22 |
| Table S12 |  |
| Number of feedback-locked trials per condition for the MLM | 25 |
| Table S13 |  |
| Trials per condition for the grand-averages | 26 |
| Table S14 |  |
| **References** | **27** |

**Power analysis**

We conducted an a priori power analysis using G*power (Version 3.1.9.2; Faul et al., 2007) for the pre-registered repeated measures analysis of variance (ANOVA) on the FRN with a rather small effect size of partial eta square = .10 and a power of 90%. This analysis yielded a necessary total sample size of *N* = 48 (i.e., 24 per group) at the standard alpha level of .05. We conducted Mixed Linear Models on the single trial EEG data instead of the traditional ANOVA and comparable studies show similar sample sizes (*N* = 20 in Weber & Bellebaum, 2024; *N* = 52 with 26 participants per group in Huvermann et al., 2025).

**Neurological and neuropsychological assessment**

In addition to the tests mentioned in the main manuscript, we used the International Cooperative Ataxia Rating Scale (ICARS; Trouillas et al., 1997), the Inventory of Non-Ataxia Signs (INAS; Jacobi et al., 2013), and the Brief Ataxia Rating Scale (BARS; Schmahmann et al., 2009) to characterize the ataxia and non-ataxia symptoms. To compare motor performance between groups, we assessed the SCAFI (Spinocerebellar Ataxia Functional Index; Schmitz-Hübsch et al., 2008) for all participants. The ABC-D (Activities-specific Balance Confidence Scale; Powell & Myers, 1995), MSWS (Multiple Sclerosis Walking Scale; Hobart et al., 2003), HADS (Hospital Anxiety and Depression Scale; Zigmond & Snaith, 1983), EQD-5 (EuroQol-5 Dimensions; EuroQol, 1990), and PHQ-9 (Patient Health Questionnaire – Brief; Kroenke et al., 2001) were used to investigate the current emotional and health state as well as the perceived general quality of life.

| Table S1: Full table of results of the neurological and neuropsychological assessment | | | | | | | | |
| --- | --- | --- | --- | --- | --- | --- | --- | --- |
|  |  | **Patients** |  |  | **Controls** |  |  |  |
| **Function and test** | | ***n*** | ***M*** | ***SD*** | ***n*** | ***M*** | ***SD*** | ***p*** |
| ***Premorbid intelligence*** | |  |  |  |  |  |  |  |
| MWT-B |  | 21 | 108.00 | 10.81 | 25 | 111.40 | 9.84 | n.s. (.282) |
| ***Severity of ataxia*** | |  |  |  |  |  |  |  |
| SARA |  | 21 | 9.17 | 3.47 | NA | NA | NA |  |
| SCAFI |  | 21 | -0.62 | 0.53 | 23 | 0.72 | 0.45 | < .001 |
| BARS |  | 21 | 11.57 | 3.56 | NA | NA | NA |  |
| ICARS |  | 21 | 25.90 | 9.64 | NA | NA | NA |  |
| ***Extracerebellar involvement*** | | |  |  |  |  |  |  |
| INAS |  | 21 | 1.10 | 1.09 | NA | NA | NA |  |
| ***Neuropsychological deficits*** | | |  |  |  |  |  |  |
| CCAS Scale |  | 21 | 1.33 | 1.46 | 25 | 1.48 | 1.76 | n.s. (.945) |
| ***Depressed mood*** | |  |  |  |  |  |  |  |
| BDI-II |  | 21 | 8.38 | 5.73 | 25 | 3.12 | 2.82 | < .001 |
| PHQ-9 |  | 21 | 6.48 | 4.41 | 25 | 1.96 | 2.19 | < .001 |
| HADS |  | 21 | 3.71 | 2.19 | 25 | 1.64 | 1.60 | =.001 |
| *depression* |  | 21 | 4.29 | 3.39 | 25 | 2.84 | 2.61 | n.s. (.133) |
| *anxiety* |  |  |  |  |  |  |  |  |
| ***Health status*** |  | 21 | 84.29 | 13.56 | 23 | 89.60 | 39.48 | < .001 |
| EQ-5D |  |  |  |  |  |  |  |  |
| *Index VAS* |  | 21 | 70.90 | 15.73 | 23 | 88.70 | 8.51 | < .001 |
| ***Motor function*** | |  |  |  |  |  |  |  |
| MSWS-12 |  | 21 | 33.19 | 10.72 | 25 | 12.00 | 0 | < .001 |
| ABC-D |  | 21 | 32.76 | 9.23 | 25 | 15.76 | 5.1 | < .001 |
| *Note.* MSWS: control group always rated the lowest item, resulting in no standard deviation. Two missing values for EQD-5 and SCAFI scores led to *n* = 23 for controls. For the test statistics (when applicable), *t*-tests for parametric and Wilcoxon rank test for non-parametric distribution were calculated. | | | | | | | | |

| **Complete inferential statistics for the MLMs reported in the main manuscript**  Table S2: Inferential statistics for the MLM analysis examining the effects of group, feedback timing, and block on accuracy | | | | | | |
| --- | --- | --- | --- | --- | --- | --- |
| Fixed Effects | | | | | | |
|  | *Est*/β | *SE* | *df* | *t* | *p* |  |
| **(Intercept)** | **71.04** | **2.31** | **44** | **30.70** | **< .001** |  |
| **feedback timing** | **-4.52** | **2.15** | **44** | **-2.11** | **.041** |  |
| group | -4.46 | 4.63 | 44 | -0.96 | .341 |  |
| **block** | **5.50** | **0.78** | **44** | **7.09** | **< .001** |  |
| feedback timing:group | 7.52 | 4.30 | 44 | 1.75 | .087 |  |
| feedback timing:block | -1.39 | 1.63 | 44 | -0.85 | .399 |  |
| group:block | -0.85 | 1.55 | 44 | -0.55 | .586 |  |
| feedback timing:group:block | -0.20 | 3.27 | 44 | -0.06 | .952 |  |
| Random Effects | | | | | | |
|  | *Variance* | *SD* | *Corr* | | |  |
| subject (Intercept) | 235.62 | 15.35 |  |  |  |  |
| subject (feedback timing) | 175.25 | 13.24 | 0.09 |  |  |  |
| subject (block) | 18.56 | 4.31 | 0.50 | -0.08 |  |  |
| subject (feedback timing:block) | 86.33 | 9.29 | 0.06 | 0.34 | -0.07 |  |
| Residual | 141.41 | 11.89 |  |  |  |  |
| Model fit | | | | | | |
|  | marginal | | | conditional | | |
| R^2^ | 0.09 | | | 0.72 | | |
| Key: *p*-values for fixed effects calculated using Satterthwaites approximations. Model equation: accuracy ~ feedback timing * group * block + (1 + feedback timing*block \| subject) | | | | | | |
| *Note*. *n*_subjects_ = 46, *n*_observations_ = 736. Bold text indicates significant effects. | | | | | | |

| Table S3: Inferential statistics for the MLM analysis examining the effects of feedback valence, response type, feedback timing, group, and block on choice switching | | | | | | |
| --- | --- | --- | --- | --- | --- | --- |
| Fixed Effects | | | | | | |
|  | *Est*/β | *SE* | *df* | *t* | *p* |  |
| **(Intercept)** | **0.19** | **0.03** | **42.52** | **6.63** | **< .001** |  |
| **feedback valence** | **-0.42** | **0.04** | **37.94** | **-9.59** | **< .001** |  |
| **response type** | **-0.34** | **0.07** | **40.37** | **-4.63** | **< .001** |  |
| feedback timing | 0.07 | 0.04 | 106.72 | 1.65 | .101 |  |
| group | 0.01 | 0.06 | 42.52 | 0.23 | .823 |  |
| **block** | **-0.06** | **0.01** | **7167.74** | **-3.98** | **< .001** |  |
| **feedback valence:response type** | **0.18** | **0.08** | **32.51** | **2.09** | **.044** |  |
| feedback valence:feedback timing | -0.02 | 0.06 | 4802.26 | -0.36 | .716 |  |
| **response type:feedback timing** | **0.16** | **0.06** | **5010.70** | **2.55** | **.011** |  |
| feedback valence:group | 0.01 | 0.09 | 37.94 | 0.13 | .896 |  |
| response type:group | 0.15 | 0.15 | 40.37 | 1.03 | .309 |  |
| feedback timing:group | -0.10 | 0.09 | 106.72 | -1.14 | .256 |  |
| feedback valence:block | -0.02 | 0.03 | 6584.24 | -0.82 | .410 |  |
| **response type:block** | **-0.12** | **0.03** | **6913.05** | **-4.08** | **< .001** |  |
| feedback timing:block | 0.02 | 0.03 | 7414.71 | 0.69 | .490 |  |
| group:block | 0.03 | 0.03 | 7167.74 | 0.94 | .347 |  |
| feedback valence:response type:feedback timing | -0.18 | 0.12 | 5356.13 | -1.50 | .133 |  |
| feedback valence:response type:group | -0.05 | 0.17 | 32.51 | -0.27 | .786 |  |
| feedback valence:feedback timing:group | -0.16 | 0.12 | 4802.26 | -1.30 | .193 |  |
| response type:feedback timing:group | -0.09 | 0.12 | 5010.70 | -0.73 | .466 |  |
| feedback valence:response type:block | 0.11 | 0.06 | 7098.91 | 1.81 | .071 |  |
| feedback valence:feedback timing:block | 0.01 | 0.06 | 6775.74 | 0.09 | .931 |  |
| response type:feedback timing:block | 0.03 | 0.06 | 7078.28 | 0.44 | .662 |  |
| feedback valence:group:block | 0.00 | 0.06 | 6584.24 | 0.02 | .981 |  |
| response type:group:block | -0.01 | 0.06 | 6913.05 | -0.12 | .904 |  |
| **feedback timing:group:block** | **-0.12** | **0.06** | **7414.71** | **-1.98** | **.048** |  |
| feedback valence:response type:feedback timing:group | 0.25 | 0.24 | 5356.13 | 1.05 | .292 |  |
| feedback valence:response type:feedback timing:block | -0.15 | 0.12 | 7324.80 | -1.29 | .197 |  |
| feedback valence:response type:group:block | -0.10 | 0.12 | 7098.91 | -0.90 | .370 |  |
| feedback valence:feedback timing:group:block | 0.02 | 0.12 | 6775.74 | 0.16 | .870 |  |
| response type:feedback timing:group:block | 0.04 | 0.12 | 7078.28 | 0.37 | .709 |  |
| feedback valence:response type:feedback timing:group:block | 0.44 | 0.23 | 7324.80 | 1.87 | .061 |  |
|  |  |  |  |  |  |  |
| Random Effects | | | | | | |
|  | *Variance* | *SD* | *Corr* | | | |
| subject (Intercept) | 0.03 | 0.16 |  |  |  |  |
| subject (response type) | 0.21 | 0.45 | 0.73 |  |  |  |
| subject (feedback timing) | 0.04 | 0.21 | -0.14 | 0.14 |  |  |
| subject (feedback valence) | 0.04 | 0.21 | 0.17 | 0.06 | 0.00 |  |
| subject (response type:feedback valence) | 0.15 | 0.39 | -0.71 | -0.56 | 0.03 | -0.60 |
| Residual | 0.79 | 0.89 |  |  |  |  |
| Model fit | | | | | | |
|  | marginal | | | conditional | | |
| R^2^ | 0.12 | | | 0.22 | | |
| Key: *p*-values for fixed effects calculated using Satterthwaites approximations. Model equation: Choice switching ~ feedback valence*response type*feedback timing*group*block + (1+response type + feedback timing + feedback valence + feedback valence:response type \| subject) | | | | | | |
| *Note*. *n*_subjects_ = 46, *n*_observations_ = 14066. Bold text indicates significant effects. | | | | | | |

**Choice switching**

For choice switching, the main effect of block was significant (β = -0.06, *t*(7167.74) = -3.98, *p* < .001). Choice switching was more frequent at the beginning of the task compared to later. This effect was further modulated by response type, as reflected in a significant interaction between response type and block (β = -0.12, *t*(6913.05) = -4.08, *p* < .001). Simple slope analysis using response type as the moderating factor revealed a non-significant effect for block (*p* = .692) for incorrect choices, indicating a constant rate of choice switching throughout the task following errors. In contrast, the effect for correct responses was significant (β = -0.11, *SE* = 0.01, *t* = -10.02, *p* < .001), with reduced choice switching in late compared to early blocks, consistent with learning. A significant two-way interaction between response type and feedback valence was present (β = 0.18, *t*(32.51) = 2.09, *p* = .044). Simple slope analysis using response type as the moderating factor revealed reduced choice switching for positive compared to negative feedback and slightly stronger choice switching for incorrect (β = -0.51, *SE* = 0.08, *t* = -6.65, *p* < .001) than for correct responses (β = -0.33, *SE* = 0.04, *t* = -8.29, *p* < .001). Also, a significant two-way interaction between response type and feedback timing (β = 0.16, *t*(5010.70) = 2.55, *p* = .011) was found. Simple slope analysis using response type as the moderating factor showed that for correct choices, the effect was significant (β = 0.14, *SE* = 0.04, *t* = 3.60, *p* < .001), indicating more choice switching for immediate compared to delayed feedback. For incorrect choices, the effect was nonsignificant (*p* = .905). All other main and interaction effects were nonsignificant (all *p*-values ≥ .061).

| Table S4: Inferential statistics for the MLM analysis examining the effects of group, feedback timing, feedback valence, unsigned PE, and learnability on single-trial FRN amplitude | | | | | | | | | | | | |  | |  |  |  |
| --- | --- | --- | --- | --- | --- | --- | --- | --- | --- | --- | --- | --- | --- | --- | --- | --- | --- |
| Fixed Effects | | | | | | | | | | | | |  | |  |  |  |
|  | *Est*/β | | *SE* | | *df* | | *t* | | *p* | | |  |  | |  |  |  |
| **(Intercept)** | **2.89** | | **0.31** | | **44.25** | | **9.43** | | **< .001** | | |  |  | |  |  |  |
| **group** | **-1.61** | | **0.61** | | **44.25** | | **-2.64** | | **.011** | | |  |  | |  |  |  |
| **feedback timing** | **-1.14** | | **0.46** | | **44.45** | | **-2.5** | | **.016** | | |  |  | |  |  |  |
| **feedback valence** | **0.65** | | **0.17** | | **43.61** | | **3.85** | | **< .001** | | |  |  | |  |  |  |
| **unsigned PE** | **0.35** | | **0.16** | | **3000.76** | | **2.22** | | **.027** | | |  |  | |  |  |  |
| learnability | -0.14 | | 0.14 | | 46.55 | | -1.06 | | .294 | | |  |  | |  |  |  |
| **group:feedback timing** | **2.06** | | **0.91** | | **44.45** | | **2.26** | | **.029** | | |  |  | |  |  |  |
| group:feedback valence | 0.22 | | 0.34 | | 43.61 | | 0.65 | | .520 | | |  |  | |  |  |  |
| **feedback timing:feedback valence** | **-0.81** | | **0.34** | | **45.29** | | **-2.34** | | **.024** | | |  |  | |  |  |  |
| group:unsigned PE | -0.05 | | 0.31 | | 3000.76 | | -0.17 | | .868 | | |  |  | |  |  |  |
| feedback timing:unsigned PE | -0.27 | | 0.31 | | 3270.32 | | -0.87 | | .386 | | |  |  | |  |  |  |
| feedback valence:unsigned PE | 0.54 | | 0.33 | | 5672.17 | | 1.61 | | .107 | | |  |  | |  |  |  |
| group:learnability | 0.11 | | 0.27 | | 46.55 | | 0.41 | | .684 | | |  |  | |  |  |  |
| feedback timing:learnability | -0.21 | | 0.26 | | 45.47 | | -0.82 | | .417 | | |  |  | |  |  |  |
| feedback valence:learnability | -0.23 | | 0.28 | | 46.26 | | -0.82 | | .417 | | |  |  | |  |  |  |
| unsigned PE:learnability | 0.02 | | 0.31 | | 2259.86 | | 0.06 | | .953 | | |  |  | |  |  |  |
| group:feedback timing:feedback valence | 0.53 | | 0.69 | | 45.29 | | 0.77 | | .446 | | |  |  | |  |  |  |
| group:feedback timing:unsigned PE | 0.09 | | 0.62 | | 3270.32 | | 0.14 | | .891 | | |  |  | |  |  |  |
| **group:feedback valence:unsigned PE** | **-1.36** | | **0.67** | | **5672.17** | | **-2.04** | | **.041** | | |  |  | |  |  |  |
| feedback timing:feedback valence:unsigned PE | 0.83 | | 0.66 | | 5410.76 | | 1.25 | | .211 | | |  |  | |  |  |  |
| group:feedback timing:learnability | 0.89 | | 0.52 | | 45.47 | | 1.7 | | .096 | | |  |  | |  |  |  |
| group:feedback valence:learnability | 0.06 | | 0.56 | | 46.26 | | 0.11 | | .910 | | |  |  | |  |  |  |
| feedback timing:feedback valence:learnability | -0.69 | | 0.39 | | 23289.59 | | -1.76 | | .079 | | |  |  | |  |  |  |
| group:unsigned PE:learnability | -0.13 | | 0.61 | | 2259.86 | | -0.21 | | .831 | | |  |  | |  |  |  |
| feedback timing:unsigned PE:learnability | 0.04 | | 0.59 | | 8058.12 | | 0.06 | | .949 | | |  |  | |  |  |  |
| feedback valence:unsigned PE:learnability | -0.63 | | 0.63 | | 1572.48 | | -1 | | .318 | | |  |  | |  |  |  |
| group:feedback timing:feedback valence:unsigned PE | -1.07 | | 1.32 | | 5410.76 | | -0.81 | | .421 | | |  |  | |  |  |  |
| group:feedback timing:feedback valence:learnability | 0.06 | | 0.78 | | 23289.59 | | 0.07 | | .941 | | |  |  | |  |  |  |
| group:feedback timing:unsigned PE:learnability | 0.11 | | 1.18 | | 8058.12 | | 0.09 | | .929 | | |  |  | |  |  |  |
| group:feedback valence:unsigned PE:learnability | 1.71 | | 1.26 | | 1572.48 | | 1.36 | | .174 | | |  |  | |  |  |  |
| feedback timing:feedback valence:unsigned PE:learnability | -1.48 | | 1.24 | | 1225.13 | | -1.19 | | .234 | | |  |  | |  |  |  |
| group:feedback timing:feedback valence:unsigned PE:learnability | 2.53 | | 2.49 | | 1225.13 | | 1.02 | | .309 | | |  |  | |  |  |  |
| Random Effects | | | | | | | | | | | | | |  | | |  |
|  | | *Variance* | | *SD* | | *Corr* | | | | | | | | | | | |
| subject (Intercept) | | 4.16 | | 2.04 | |  | |  | |  |  | | |  | | |  |
| subject (feedback timing) | | 9.01 | | 3.00 | | -0.03 | |  | |  |  | | |  | | |  |
| subject (feedback valence) | | 0.87 | | 0.94 | | 0.25 | | 0.22 | |  |  | | |  | | |  |
| subject (learnability) | | 0.39 | | 0.62 | | 0.24 | | -0.31 | | 0.12 |  | | |  | | |  |
| subject (feedback timing:feedback valence) | | 3.65 | | 1.91 | | 0.06 | | 0.48 | | 0.15 | 0.00 | | |  | | |  |
| subject (feedback valence:learnability) | | 1.83 | | 1.35 | | 0.02 | | 0.12 | | -0.29 | 0.28 | | | 0.07 | | |  |
| subject (feedback timing:learnability) | | 1.35 | | 1.16 | | -0.20 | | -0.04 | | -0.57 | -0.22 | | | 0.44 | | | 0.22 |
| Residual | | 53.16 | | 7.29 | |  | |  | |  |  | | |  | | |  |
| Model fit | | | | | | | | | | | | | |  | | |  |
|  | | marginal | | | | | | conditional | | | | | |  | | |  |
| R^2^ | | 0.02 | | | | | | 0.14 | | | | | |  | | |  |
| Key: *p*-values for fixed effects calculated using Satterthwaites approximations. Model equation: FRN ~ 1 + group * feedback timing * feedback valence * unsigned PE * learnability + (1 + feedback timing * feedback valence + learnability + feedback valence:learnability + feedback timing:learnability \| subject) | | | | | | | | | | | | | |  | | |  |
| *Note*. *n*_subjects_ = 46, *n*_observations_ = 27111. Bold text indicates significant effects. | | | | | | | | | | | | | |  | | |  |

**FRN**

The main effect of feedback timing was significant (β = -1.14, *t*(44.45) = -2.50, *p* = .016). The FRN was more negative for delayed (*M* = 2.28 µV, *SD* = 8.10 µV) compared to immediate feedback (*M* = 3.52 µV, *SD* = 7.53 µV). Also, a significant interaction between feedback valence and feedback timing was found (β = -0.81, *t*(45.29) = -2.34, *p* = .024). Simple slope analysis revealed that the effect of feedback valence was significant for immediate feedback (β = 1.05, *SE* = 0.23, *t* = 4.61, *p* < .001), indicating a more negative FRN for negative (*M* = 3.05 µV, *SD* = 7.52 µV) compared to positive feedback (*M* = 3.89 µV, *SD* = 7.53 µV). For delayed feedback, the effect was nonsignificant (*p* = .526). Importantly, the main effect of unsigned PE was also significant (β = 0.35, *t*(3000.76) = 2.22, *p* = .027), reflecting a less negative FRN for higher unsigned PE. All other main and interaction effects were nonsignificant (all *p*-values ≥ .079).

| Table S5: Inferential statistics for the MLM analysis examining the effects of group, feedback timing, feedback valence, unsigned PE, and learnability on single-trial P3a mean amplitude | | | | | | |
| --- | --- | --- | --- | --- | --- | --- |
| Fixed Effects | | | | | | |
|  | *Est*/β | *SE* | *df* | *t* | *p* |  |
| **(Intercept)** | **5.31** | **0.35** | **44.23** | **15.13** | **< .001** |  |
| group | -1.23 | 0.70 | 44.23 | -1.75 | .087 |  |
| feedback timing | -0.46 | 0.67 | 44.24 | -0.68 | .503 |  |
| **unsigned PE** | **0.56** | **0.15** | **3715.93** | **3.81** | **< .001** |  |
| **feedback valence** | **0.57** | **0.14** | **45.88** | **4.03** | **< .001** |  |
| **learnability** | **-0.43** | **0.10** | **26984.07** | **-4.45** | **< .001** |  |
| group:feedback timing | 2.25 | 1.35 | 44.24 | 1.67 | .102 |  |
| group:unsigned PE | -0.50 | 0.29 | 3715.93 | -1.71 | .087 |  |
| feedback timing:unsigned PE | -0.13 | 0.28 | 17588.57 | -0.47 | .636 |  |
| group:feedback valence | 0.02 | 0.28 | 45.88 | 0.06 | .954 |  |
| feedback timing:feedback valence | -0.13 | 0.19 | 26814.87 | -0.66 | .507 |  |
| **unsigned PE:feedback valence** | **1.01** | **0.32** | **26811.17** | **3.15** | **.002** |  |
| group:learnability | 0.13 | 0.19 | 26984.07 | 0.68 | .498 |  |
| feedback timing:learnability | 0.15 | 0.19 | 27005.27 | 0.78 | .435 |  |
| **unsigned PE:learnability** | **0.64** | **0.28** | **18997.71** | **2.29** | **.022** |  |
| feedback valence:learnability | -0.14 | 0.19 | 26281.89 | -0.72 | .469 |  |
| group:feedback timing:unsigned PE | -0.14 | 0.56 | 17588.57 | -0.25 | .806 |  |
| group:feedback timing:feedback valence | -0.54 | 0.39 | 26814.87 | -1.39 | .164 |  |
| **group:unsigned PE:feedback valence** | **-2.53** | **0.64** | **26811.17** | **-3.95** | **< .001** |  |
| feedback timing:unsigned PE:feedback valence | 0.90 | 0.64 | 26841.98 | 1.40 | .162 |  |
| group:feedback timing:learnability | 0.44 | 0.39 | 27005.27 | 1.14 | .253 |  |
| **group:unsigned PE:learnability** | **-1.18** | **0.56** | **18997.71** | **-2.10** | **.036** |  |
| feedback timing:unsigned PE:learnability | 0.52 | 0.56 | 21561.82 | 0.93 | .350 |  |
| group:feedback valence:learnability | -0.29 | 0.39 | 26281.89 | -0.74 | .459 |  |
| feedback timing:feedback valence:learnability | -0.14 | 0.39 | 26943.76 | -0.36 | .718 |  |
| unsigned PE:feedback valence:learnability | -0.12 | 0.58 | 26928.68 | -0.21 | .830 |  |
| group:feedback timing:unsigned PE:feedback valence | -1.20 | 1.28 | 26841.98 | -0.94 | .349 |  |
| group:feedback timing:unsigned PE:learnability | -0.23 | 1.12 | 21561.82 | -0.20 | .840 |  |
| group:feedback timing:feedback valence:learnability | -1.18 | 0.77 | 26943.76 | -1.53 | .126 |  |
| group:unsigned PE:feedback valence:learnability | 1.55 | 1.16 | 26928.68 | 1.33 | .184 |  |
| feedback timing:unsigned PE:feedback valence:learnability | -2.14 | 1.16 | 27038.65 | -1.85 | .065 |  |
| group:feedback timing:unsigned PE:feedback valence:learnability | 4.44 | 2.32 | 27038.65 | 1.91 | .056 |  |
| Random Effects | | | | | | |
|  | *Variance* | *SD* | *Corr* | | | |
| subject (Intercept) | 5.52 | 2.35 |  |  |  |  |
| subject (feedback timing) | 20.30 | 4.51 | -0.1 |  |  |  |
| subject (feedback valence) | 0.48 | 0.69 | 0.02 | 0.05 |  |  |
| Residual | 52.28 | 7.23 |  |  |  |  |
| Model fit |  |  |  |  |  |  |
|  | marginal | | | conditional | | |
| R^2^ | 0.02 | | | 0.18 | | |
| Key: *p*-values for fixed effects calculated using Satterthwaites approximations. Model equation: P3a ~ 1 + group * feedback timing * unsigned PE * feedback valence * learnability + (1 + feedback timing + feedback valence \| subject) | | | | | | |
| *Note*. *n*_subjects_ = 46, *n*_observations_ = 27111. Bold text indicates significant effects. | | | | | | |

**P3a**

For the P3a, we found a significant main effect of the unsigned PE (β = 0.56, *t*(3715.93) = 3.81, *p* < .001). The estimate of the effect indicated a more positive P3a for higher unsigned PEs. Also, the main effect of learnability was significant (β = -0.43, *t*(26984.07) = -4.45, *p* < .001). The P3a was more positive for unlearnable (*M* = 5.52 µV, *SD* = 8.00 µV) compared to learnable trials (*M* = 4.98 µV, *SD* = 7.92 µV). Importantly, there was a significant interaction between the unsigned PE and feedback valence (β = 1.01, *t*(26811.17) = 3.15, *p* = .002). Simple slope analysis demonstrated a significant effect for positive feedback only (β = 1.12, *SE* = 0.21, *t* = 5.43, *p* < .001), with increased P3a for higher unsigned PEs. Interestingly, there was also a significant interaction between the unsigned PE and learnability (β = 0.64, *t*(18997.71) = 2.29, *p* = .022). Simple slope analysis revealed a significant effect for learnable trials only (β = 0.99, *SE* = 0.21, *t* = 4.60, *p* < .001), with increasing P3a amplitudes with higher unsigned PE across groups. The effect for unlearnable trials was not significant (*p* = .091). All other main and interaction effects (all *p*-values ≥ .056) were non-significant.

| Table S6: Inferential statistics for the MLM analysis examining the effects of group, feedback timing, feedback valence, unsigned PE, and learnability on single-trial P3b mean amplitude | | | | | | |
| --- | --- | --- | --- | --- | --- | --- |
| Fixed Effects | | | | | | |
|  | *Est*/β | *SE* | *df* | *t* | *p* |  |
| **(Intercept)** | **6.34** | **0.40** | **44.15** | **15.68** | **< .001** |  |
| group | -1.25 | 0.81 | 44.15 | -1.54 | .130 |  |
| **feedback timing** | **2.18** | **0.55** | **44.35** | **3.95** | **< .001** |  |
| **feedback valence** | **0.74** | **0.18** | **44.21** | **4.06** | **< .001** |  |
| **unsigned PE** | **0.54** | **0.22** | **44.89** | **2.47** | **.017** |  |
| **learnability** | **-0.34** | **0.11** | **26836.04** | **-3.21** | **.001** |  |
| group:feedback timing | 1.73 | 1.10 | 44.35 | 1.57 | .123 |  |
| group:feedback valence | 0.13 | 0.36 | 44.21 | 0.36 | .720 |  |
| feedback timing:feedback valence | -0.14 | 0.22 | 2749.54 | -0.64 | .520 |  |
| group:unsigned PE | -0.24 | 0.44 | 44.89 | -0.55 | .587 |  |
| feedback timing:unsigned PE | -0.02 | 0.41 | 49.75 | -0.04 | .970 |  |
| **feedback valence:unsigned PE** | **1.06** | **0.35** | **25380.00** | **3.01** | **.003** |  |
| group:learnability | -0.08 | 0.21 | 26836.04 | -0.39 | .700 |  |
| feedback timing:learnability | 0.17 | 0.21 | 26844.79 | 0.78 | .433 |  |
| feedback valence:learnability | -0.15 | 0.22 | 9847.07 | -0.71 | .476 |  |
| unsigned PE:learnability | 0.23 | 0.31 | 10292.31 | 0.73 | .466 |  |
| group:feedback timing:feedback valence | 0.02 | 0.44 | 2749.54 | 0.05 | .963 |  |
| group:feedback timing:unsigned PE | 0.30 | 0.81 | 49.75 | 0.37 | .711 |  |
| **group:feedback valence:unsigned PE** | **-2.00** | **0.70** | **25380.00** | **-2.84** | **.004** |  |
| feedback timing:feedback valence:unsigned PE | 0.85 | 0.70 | 23933.17 | 1.22 | .224 |  |
| group:feedback timing:learnability | 0.37 | 0.42 | 26844.79 | 0.86 | .390 |  |
| group:feedback valence:learnability | 0.26 | 0.43 | 9847.07 | 0.60 | .549 |  |
| feedback timing:feedback valence:learnability | 0.10 | 0.43 | 10197.11 | 0.23 | .816 |  |
| group:unsigned PE:learnability | 0.02 | 0.63 | 10292.31 | 0.04 | .971 |  |
| feedback timing:unsigned PE:learnability | 0.25 | 0.63 | 9240.63 | 0.40 | .688 |  |
| feedback valence:unsigned PE:learnability | 1.12 | 0.64 | 24765.22 | 1.75 | .080 |  |
| **group:feedback timing:feedback valence:unsigned PE** | **-3.79** | **1.40** | **23933.17** | **-2.71** | **.007** |  |
| group:feedback timing:feedback valence:learnability | 0.04 | 0.86 | 10197.11 | 0.05 | .961 |  |
| group:feedback timing:unsigned PE:learnability | 2.11 | 1.26 | 9240.63 | 1.68 | .093 |  |
| group:feedback valence:unsigned PE:learnability | -1.32 | 1.28 | 24765.22 | -1.04 | .300 |  |
| feedback timing:feedback valence:unsigned PE:learnability | -1.69 | 1.28 | 24313.76 | -1.33 | .185 |  |
| group:feedback timing:feedback valence:unsigned PE:learnability | 1.82 | 2.55 | 24313.76 | 0.71 | .475 |  |
| Random Effects | | | | | | |
|  | *Variance* | *SD* | *Corr* | | | |
| subject (Intercept) | 7.34 | 2.71 |  |  |  |  |
| subject (feedback timing) | 13.36 | 3.66 | 0.19 |  |  |  |
| subject (feedback valence) | 0.94 | 0.97 | 0.16 | 0.28 |  |  |
| subject (unsigned PE) | 0.91 | 0.96 | -0.08 | 0.47 | 0.25 |  |
| subject (feedback timing:unsigned PE) | 3.01 | 1.73 | 0.33 | 0.35 | 0.32 | -0.08 |
| Residual | 62.42 | 7.90 |  |  |  |  |
| Model fit | | | | | | |
|  | marginal | | | conditional | | |
| R^2^ | 0.03 | | | 0.17 | | |
| Key: *p*-values for fixed effects calculated using Satterthwaites approximations. Model equation: P3b ~ 1 + group * feedback timing * feedback valence * unsigned PE * learnability + (1 + feedback timing + feedback valence + unsigned PE + feedback timing:unsigned PE \| subject) | | | | | | |
| *Note*. *n*_subjects_ = 46, *n*_observations_ = 27111. Bold text indicates significant effects. | | | | | | |

**P3b main model**

The analysis of the main model (Table S6) revealed a significant main effect of feedback timing (β = 2.18, *t*(44.35) = 3.95, *p* < .001), indicating that the P3b was more pronounced for delayed (*M* = 7.30 µV, *SD* = 9.08 µV) compared to immediate feedback (*M* = 5.23 µV, *SD* = 9.08 µV). In addition, the main effect of feedback valence was significant (β = 0.74, *t*(44.21) = 4.06, *p* < .001), with higher P3b amplitudes for positive (*M* = 6.51 µV, *SD* = 8.59 µV) compared to negative feedback (*M* = 5.93 µV, *SD* = 8.71 µV). Also, the main effect of the unsigned PE was significant (β = 0.54, *t*(44.89) = 2.47, *p* = .017), revealing increased P3b amplitudes with higher unsigned PEs. Last, the main effect of learnability was significant (β = -0.34, *t*(26836.04) = -3.21, *p* < .001), with decreased P3b amplitudes for learnable (*M* = 6.03 µV, *SD* = 8.71 µV) compared to unlearnable trials (*M* = 6.49 µV, *SD* = 8.57 µV).

| Table S7: Inferential statistics for the separate model for patients on P3b mean amplitude to resolve the four-way interaction of the main model | | | | | | |
| --- | --- | --- | --- | --- | --- | --- |
| Fixed Effects | | | | | | |
|  | *Est*/β | *SE* | *df* | *t* | *p* |  |
| **(Intercept)** | **5.72** | **0.60** | **20.00** | **9.55** | **< .001** |  |
| **feedback timing** | **3.00** | **0.62** | **20.06** | **4.89** | **< .001** |  |
| **feedback valence** | **0.74** | **0.22** | **19.45** | **3.39** | **.003** |  |
| unsigned PE | 0.51 | 0.24 | 15.65 | 2.12 | .050 |  |
| feedback timing:feedback valence | -0.14 | 0.35 | 20.6 | -0.39 | .700 |  |
| feedback timing:unsigned PE | -0.07 | 0.41 | 429.95 | -0.18 | .856 |  |
| feedback valence:unsigned PE | 0.39 | 0.46 | 9929.39 | 0.85 | .398 |  |
| feedback timing:feedback valence:unsigned PE | -1.25 | 0.91 | 8349.10 | -1.37 | .170 |  |
| Random Effects | | | | | | |
|  | *Variance* | *SD* | *Corr* | | | |
| subject (Intercept) | 7.43 | 2.73 |  |  |  |  |
| subject (feedback timing) | 7.47 | 2.73 | 0.45 |  |  |  |
| subject (feedback valence) | 0.59 | 0.77 | -0.30 | 0.17 |  |  |
| subject (unsigned PE) | 0.25 | 0.50 | -0.01 | 0.45 | 0.33 |  |
| subject (feedback timing:feedback valence) | 1.08 | 1.04 | -0.14 | 0.60 | 0.04 | 0.05 |
| Residual | 54.41 | 7.38 |  |  |  |  |
| Model fit | | | | | | |
|  | marginal | | | conditional | | |
| R^2^ | 0.04 | | | 0.18 | | |
| Key: *p*-values for fixed effects calculated using Satterthwaites approximations. Model equation: P3b ~ 1 + feedback timing * feedback valence * unsigned PE * learnability + (1 + feedback timing*feedback valence + unsigned PE \| subject) | | | | | | |
| *Note*. *n*_subjects_ = 21, *n*_observations_ = 12697. Bold text indicates significant effects. | | | | | | |

**P3b patient group model**

For patients, the main effects of feedback timing (β = 3.00, *t*(20.06) = 4.89, *p* < .001) and feedback valence (β = 0.74, *t*(19.45) = 3.39, *p* = .003) were significant. P3b amplitudes were more positive for delayed feedback (*M* = 7.21 µV, *SD* = 8.49 µV) compared to immediate feedback (*M* = 4.18 µV, *SD* = 7.44 µV). Additionally, the P3b was more positive for positive feedback (*M* = 6.09 µV, *SD* = 8.05 µV) compared to negative feedback (*M* = 5.20 µV, *SD* = 8.20 µV). All other main and interaction effects for this model were non-significant (all *p*-values ≥ .050).

| Table S8: Inferential statistics for the separate model for controls on P3b mean amplitude to resolve the four-way interaction of the main model | | | | | | |
| --- | --- | --- | --- | --- | --- | --- |
| Fixed Effects | | | | | | |
|  | *Est*/β | *SE* | *df* | *t* | *p* |  |
| **(Intercept)** | **6.94** | **0.54** | **24.05** | **12.83** | **< .001** |  |
| feedback timing | 1.36 | 0.85 | 24.14 | 1.60 | .122 |  |
| feedback valence | 0.54 | 0.29 | 24.52 | 1.88 | .072 |  |
| **unsigned PE** | **0.70** | **0.23** | **3307.36** | **3.12** | **.002** |  |
| feedback timing:feedback valence | 0.07 | 0.52 | 24.27 | 0.14 | .894 |  |
| feedback timing:unsigned PE | -0.02 | 0.45 | 2652.38 | -0.05 | .958 |  |
| **feedback valence:unsigned PE** | **2.13** | **0.47** | **13725.59** | **4.49** | **< .001** |  |
| **feedback timing:feedback valence:unsigned PE** | **2.42** | **0.95** | **13684.19** | **2.55** | **.011** |  |
| Random Effects | | | | | | |
|  | *Variance* | *SD* | *Corr* | | | |
| subject (Intercept) | 7.18 | 2.68 |  |  |  |  |
| subject (feedback timing) | 17.41 | 4.17 | 0.03 |  |  |  |
| subject (feedback valence) | 1.54 | 1.24 | 0.45 | 0.20 |  |  |
| subject (feedback timing:feedback valence) | 4.46 | 2.11 | 0.50 | 0.19 | 0.13 |  |
| Residual | 69.49 | 8.34 |  |  |  |  |
| Model fit | | | | | | |
|  | marginal | | | conditional | | |
| R^2^ | 0.01 | | | 0.16 | | |
| Key: *p*-values for fixed effects calculated using Satterthwaites approximations. Model equation: P3b ~ 1 + feedback timing * feedback valence * unsigned PE + (1 + feedback timing*feedback valence \| subject) | | | | | | |
| *Note*. *n*_subjects_ = 25, *n*_observations_ = 14414. Bold text indicates significant effects. | | | | | | |

**P3b control group model**

For controls, the main effect of unsigned PE was significant (β = 0.70, *t*(3307.36) = 3.12, *p* = .002, with higher P3b amplitudes for higher unsigned PE.

**Homogeneity check in the patient sample**


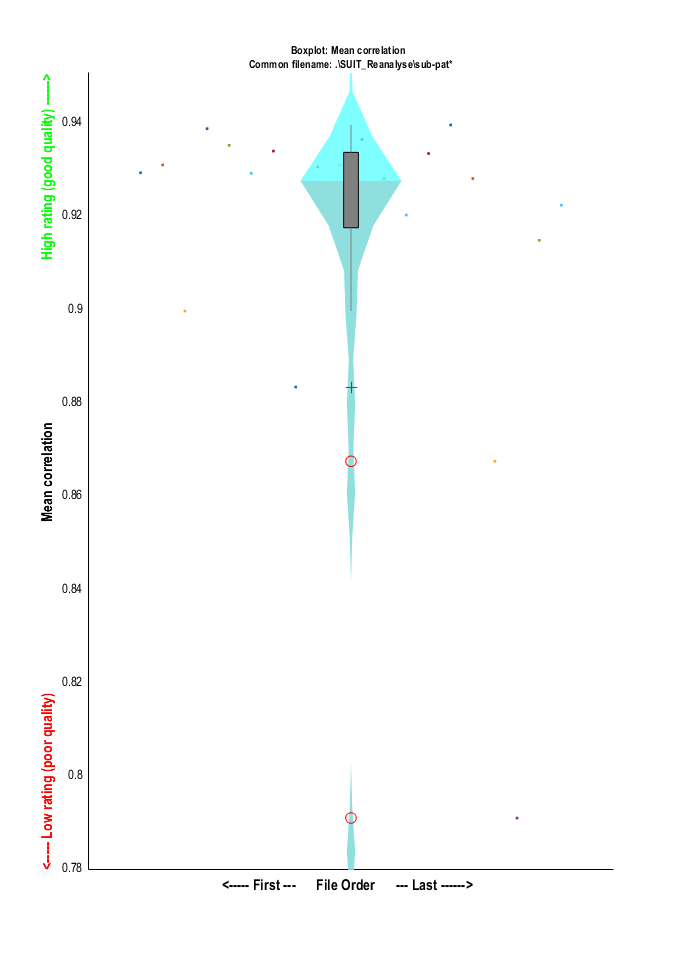


Figure S1. Boxplot of the homogeneity analysis of the patient sample. Two extreme outliers were identified who shared the same diagnosis (SCAR10, sub-pat-23 and sub-pat24).


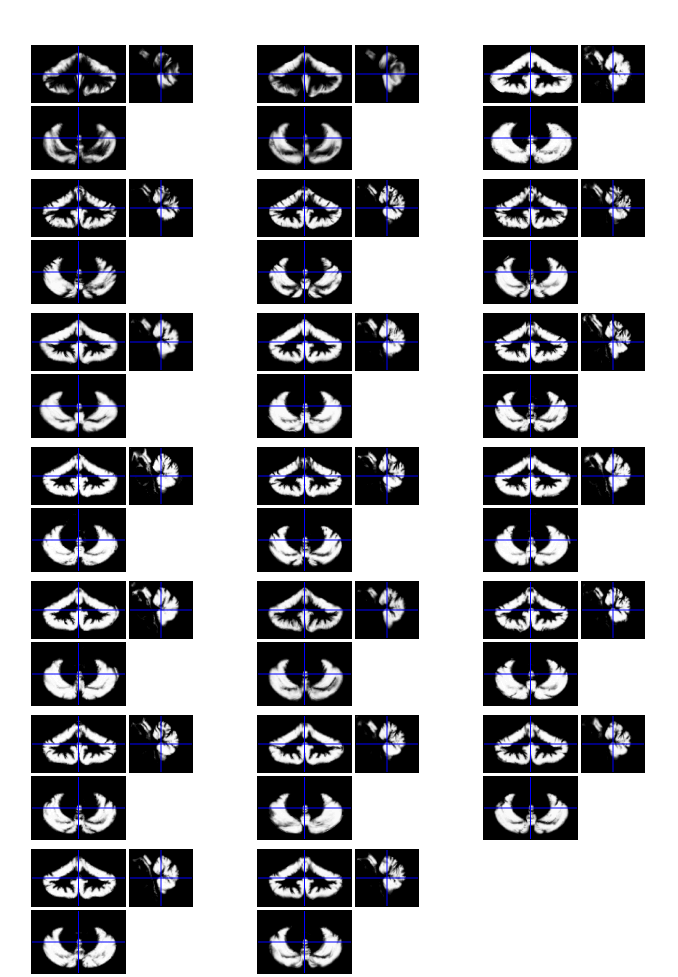


Figure S2. Visual inspection of cerebellar GMV in SUIT-space for the patient sample. The first two images show massive atrophy in sub-pat-24 and sub-pat-23.

**VBM analyses: Complete cluster lists**

| Table S9. Summary of the whole-brain between-subjects contrast control > patient for gray matter volume. Note that the gray matter volume reduction in patients was limited to the cerebellum, there were no extracerebellar clusters. | | | | | | | | |
| --- | --- | --- | --- | --- | --- | --- | --- | --- |
| index | location | side | x | y | z | cluster size^1^ | peak_p_FWE | t_peak_ |
| 1 | IV, V | vermal | 4.5 | -49.5 | -18 | 39476 | *p* < .001 | 17.97 |
|  | IV, V | left | -12 | -43.5 | -22.5 |  | *p* < .001 | 17.83 |
|  | VI | left | -25.5 | -64.5 | -25.5 |  | *p* < .001 | 16.81 |
|  | VIII | right | 9 | -66 | -30 |  | *p* < .001 | 16.75 |
|  | VIII | vermal | -4.5 | -64.5 | -28.5 |  | *p* < .001 | 16.16 |
|  | VIII | left | -7.5 | -66 | -36 |  | *p* < .001 | 15.48 |
| *Note.* ^1^cluster size in voxel. MNI-space labelled according to the Automated anatomical labelling atlas 3 (Rolls et al., 2020). | | | | | | | | |

| Table S10. Summary of the SUIT between-subjects contrast control > patient for gray matter volume - uncorrected | | | | | | | | |
| --- | --- | --- | --- | --- | --- | --- | --- | --- |
| index | location | side | x | y | z | cluster size^1^ | peak_p_uncorr | t_peak_ |
| 1 | VI | right | 29 | -38 | -35 | 66066 | *p* < .001 | 11.43 |
|  | VIIb | right | 35 | -43 | -44 |  | *p* < .001 | 10.13 |
|  | Crus I | left | -39 | -67 | -36 |  | *p* < .001 | 10.00 |
|  | Crus II | left | -8 | -80 | -39 |  | *p* < .001 | 9.80 |
|  | I-IV | right | 11 | -44 | -25 |  | *p* < .001 | 9.52 |
|  | IX | right | 10 | -50 | -46 |  | *p* < .001 | 9.46 |
| 2 | I-IV | right | 3 | -55 | 0 | 52 | .024 | 5.98 |
| 3 | VI | right | 32 | -69 | -23 | 24 | .785 | 4.37 |
| 4 | VI | right | 8 | -69 | -14 | 10 | .897 | 4.20 |
| 5 | Crus II | right | 9 | -86 | -31 | 5 | .974 | 4.00 |
| 6 | Crus I | left | -49 | -61 | -42 | 6 | .993 | 3.86 |
| 7 | VIIb | left | -37 | -63 | -55 | 6 | 1.000 | 3.58 |
| 8 | VI | vermal | -4 | -78 | -24 | 1 | 1.000 | 3.55 |
| 9 | I-IV | left | -9 | -39 | -12 | 1 | 1.000 | 3.43 |
| 10 | Crus II | right | 34 | -80 | -42 | 1 | 1.000 | 3.38 |
| 11 | Crus II | right | 5 | -82 | -32 | 1 | 1.000 | 3.32 |
| *Note.* ^1^cluster size in voxel. ^2^Clusterspread: right Crus I (5760), right Crus II (5313), left Crus II (4774), left Crus I (4445), left VI (3985), right VIIb (3647), right VI (3593), right VIIIa (3466), left VIIIa (3250), left VIIIb (2941), right VIIIb (2876), left V (2814), right I-IV (2773), right V (2684), right IX (2547), left I-IV (2415), left VIIb (2364), left IX (2023), vermal VI (1246), vermal VIIIa (1050), white matter (538), vermal IX (522), vermal VIIIb (498), vermal VIIb (239), vermal Crus II (208), vermal X (81), right dentate nuc. (8), right X (6). | | | | | | | | |

| Table S11. Summary of the between-subjects contrast control > patient for gray matter volume - FWE-corrected | | | | | | | | |
| --- | --- | --- | --- | --- | --- | --- | --- | --- |
| index | location | side | x | y | z | cluster size^1^ | peak_p_FWE | t_peak_ |
| 1 | Extended cluster^2^ | |  |  |  |  |  |  |
|  | VI | right | 29 | -38 | -35 | 11869 | *p* < .001 | 11.43 |
|  | VIIb | right | 35 | -43 | -44 |  | *p* < .001 | 10.13 |
|  | Crus I | left | -39 | -67 | -36 |  | *p* < .001 | 10.00 |
|  | Crus II | left | -8 | -80 | -39 |  | *p* < .001 | 9.80 |
|  | I-IV | right | 11 | -44 | -25 |  | *p* < .001 | 9.52 |
|  | IX | right | 10 | -50 | -46 |  | *p* < .001 | 9.46 |
| 2 | VIIIb | left | -18 | -53 | -49 | 42 | *p* < .001 | 8.30 |
| 3 | VIIIa | left | -22 | -58 | -46 | 51 | *p* < .001 | 7.96 |
| 4 | VIIIb | left | -10 | -53 | -60 | 52 | *p* < .001 | 7.63 |
| 5 | Crus I | right | 29 | -76 | -33 | 41 | *p* < .001 | 7.36 |
| 6 | Crus I | right | 20 | -79 | -30 | 23 | *p* < .001 | 7.28 |
| 7 | Crus II | left | -30 | -69 | -42 | 109 | .001 | 7.07 |
| 8 | VIIb | right | 29 | -64 | -47 | 41 | .001 | 7.07 |
| 9 | VI | left | -29 | -56 | -34 | 11 | .002 | 6.99 |
| 10 | IX | left | -9 | -56 | -37 | 12 | .002 | 6.82 |
| 11 | Crus I | left | -35 | -55 | -35 | 31 | .004 | 6.68 |
| 12 | IX | left | -11 | -53 | -41 | 3 | .009 | 6.35 |
| 13 | VI | left | -13 | -68 | -25 | 9 | .009 | 6.34 |
| 14 | VI | vermal | -1 | -71 | -15 | 5 | .011 | 6.28 |
| 15 | VIIIa | left | -15 | -67 | -44 | 7 | .014 | 6.19 |
| 16 | VI | left | -17 | -68 | -31 | 4 | .015 | 6.16 |
| 17 | VIIb | left | -14 | -70 | -43 | 6 | .018 | 6.08 |
| 18 | VIIb | left | -23 | -65 | -47 | 2 | .023 | 6.00 |
| 19 | I-IV | right | 3 | -55 | 0 | 2 | .024 | 5.98 |
| 20 | V | right | 9 | -59 | -20 | 1 | .025 | 5.97 |
| 21 | V | right | 17 | -48 | -24 | 3 | .026 | 5.95 |
| 22 | VIIb | left | -33 | -57 | -48 | 1 | .026 | 5.95 |
| 23 | Crus I | left | -17 | -77 | -30 | 3 | .026 | 5.95 |
| 24 | VI | left | -25 | -62 | -27 | 6 | .027 | 5.93 |
| 25 | I-IV | right | 3 | -40 | -19 | 1 | .027 | 5.93 |
| 26 | Crus II | left | -38 | -63 | -43 | 4 | .028 | 5.91 |
| 27 | VI | vermal | 2 | -59 | -27 | 2 | .032 | 5.87 |
| 28 | I-IV | right | 3 | -56 | -18 | 2 | .032 | 5.87 |
| 29 | V | right | 9 | -62 | -19 | 6 | .032 | 5.86 |
| 30 | white matter |  | 8 | -40 | -21 | 1 | .035 | 5.84 |
| 31 | IX | left | -10 | -55 | -40 | 1 | .035 | 5.84 |
| 32 | Crus I | left | -27 | -69 | -38 | 3 | .036 | 5.82 |
| 33 | I-IV | right | 4 | -41 | -21 | 2 | .038 | 5.80 |
| 34 | VIIIa | right | 33 | -52 | -56 | 2 | .039 | 5.80 |
| 35 | V | right | 8 | -60 | -19 | 1 | .039 | 5.79 |
| 36 | VI | left | -20 | -64 | -26 | 1 | .040 | 5.79 |
| 37 | VI | left | -17 | -59 | -24 | 2 | .043 | 5.76 |
| 38 | V | right | 25 | -40 | -28 | 1 | .044 | 5.75 |
| 39 | VI | left | -5 | -70 | -24 | 1 | .047 | 5.72 |
| 40 | VIIb | left | -26 | -64 | -45 | 2 | .048 | 5.71 |
| 41 | IX | right | 8 | -54 | -37 | 1 | .049 | 5.71 |
| 42 | VI | right | 19 | -66 | -25 | 1 | .049 | 5.70 |
| *Note.* ^1^cluster size in voxel. ^2^Clusterspread: right Crus I (1452), right Crus II (1401), left Crus II (872), right I-IV (828), right IX (742), left I-IV (706), left Crus I (677), left IX (563), left VIIIb (474), right VIIb (459), right VIIIb (423), left VI (421), vermal VIIIa (418), right VI (407), right VIIIa (407), vermal VI (341), left V (314), right V (174), left VIIIa (164), left VIIb (147), vermal VIIIb (135), vermal VIIb (123), vermal IX (119), vermal Crus II (53), white matter (34), vermal X (15). | | | | | | | | |

| Table S12. Summary of the negative correlation on the GMV for the FRN in patients - uncorrected | | | | | | | | |
| --- | --- | --- | --- | --- | --- | --- | --- | --- |
| index | location | side | x | y | z | cluster size^1^ | peak_p_uncorr | t_peak_ |
| 1 | VIIIa | left | -27 | -45 | -45 | 63 | .488 | 6.13 |
| 2 | VIIIb | left | -25 | -39 | -51 | 51 | .559 | 5.97 |
| 3 | VI | left | -16 | -66 | -30 | 38 | .567 | 5.95 |
| 4 | Crus II | left | -43 | -50 | -45 | 63 | .615 | 5.85 |
| 5 | Crus II | right | 32 | -80 | -50 | 24 | .763 | 5.53 |
| 6 | Crus II | left | -16 | -76 | -43 | 115 | .827 | 5.37 |
| 7 | Extended cluster | |  |  |  |  |  |  |
|  | Crus II | right | 18 | -76 | -43 | 249 | .856 | 5.30 |
|  | Crus II | right | 28 | -72 | -47 |  | .956 | 4.93 |
| 8 | VI | left | -24 | -62 | -28 | 33 | .858 | 5.29 |
| 9 | VIIIb | left | -21 | -48 | -59 | 2 | .872 | 5.25 |
| 10 | Extended cluster | |  |  |  |  |  |  |
|  | Crus I | left | -35 | -65 | -38 | 266 | .881 | 5.23 |
|  | Crus I | left | -45 | -70 | -40 |  | .977 | 4.78 |
|  | Crus I | left | -45 | -61 | -40 |  | 1.000 | 4.18 |
| 11 | V | right | 1 | -64 | -8 | 33 | .922 | 5.09 |
| 12 | Crus II | left | -24 | -84 | -46 | 67 | .936 | 5.03 |
| 13 | Crus II | right | 10 | -86 | -35 | 14 | .954 | 4.94 |
| 14 | VIIIb | left | -12 | -42 | -53 | 79 | .955 | 4.94 |
| 15 | Extended cluster | |  |  |  |  |  |  |
|  | Crus I | right | 35 | -67 | -32 | 103 | .965 | 4.88 |
|  | Crus I | right | 40 | -61 | -30 |  | .999 | 4.27 |
| 16 | Extended cluster | |  |  |  |  |  |  |
|  | Crus II | left | -27 | -74 | -48 | 620 | .966 | 4.87 |
|  | VIIb | left | -36 | -57 | -49 |  | .967 | 4.86 |
|  | VIIb | left | -32 | -64 | -51 |  | .969 | 4.85 |
|  | VIIb | left | -36 | -47 | -46 |  | .999 | 4.30 |
|  | VIIb | left | -20 | -68 | -49 |  | 1.000 | 4.24 |
| 17 | I-IV | right | 1 | -56 | -19 | 3 | .974 | 4.81 |
| 18 | VI | right | 39 | -59 | -26 | 23 | .986 | 4.69 |
| 19 | Crus II | left | -37 | -72 | -46 | 32 | .987 | 4.68 |
| 20 | I-IV | right | 2 | -49 | -17 | 27 | .988 | 4.66 |
| 21 | VIIIa | left | -32 | -39 | -47 | 21 | .993 | 4.58 |
| 22 | I-IV | left | -18 | -34 | -21 | 5 | .993 | 4.58 |
| 23 | Crus II | left | -10 | -84 | -33 | 10 | .993 | 4.57 |
| 24 | Extended cluster | |  |  |  |  |  |  |
|  | Crus I | right | 51 | -50 | -41 | 94 | .993 | 4.57 |
|  | Crus I | right | 49 | -61 | -43 |  | .998 | 4.40 |
| 25 | Crus I | right | 31 | -62 | -36 | 10 | .995 | 4.52 |
| 26 | IX | vermal | -1 | -59 | -43 | 33 | .995 | 4.51 |
| 27 | VIIb | vermal | 0 | -65 | -30 | 16 | .996 | 4.50 |
| 28 | Crus II | right | 9 | -84 | -41 | 17 | .996 | 4.48 |
| 29 | I-IV | right | 24 | -33 | -26 | 14 | .997 | 4.45 |
| 30 | Crus II | right | 44 | -49 | -46 | 7 | .998 | 4.41 |
| 31 | I-IV | left | -7 | -46 | -11 | 10 | .998 | 4.40 |
| 32 | VIIIa | vermal | 3 | -67 | -41 | 27 | .999 | 4.34 |
| 33 | IX | right | 3 | -53 | -48 | 17 | .999 | 4.33 |
| 34 | V | left | 0 | -61 | -5 | 3 | .999 | 4.30 |
| 35 | Crus I | right | 36 | -57 | -31 | 16 | .999 | 4.28 |
| 36 | Crus II | left | -5 | -88 | -34 | 2 | .999 | 4.28 |
| 37 | IX | right | 7 | -61 | -47 | 8 | .999 | 4.28 |
| 38 | V | left | -19 | -38 | -24 | 12 | .999 | 4.27 |
| 39 | Crus II | left | -13 | -89 | -38 | 17 | .999 | 4.26 |
| 40 | V | left | -21 | -34 | -25 | 15 | 1.000 | 4.23 |
| 41 | VI | vermal | 1 | -76 | -17 | 1 | 1.000 | 4.22 |
| 42 | IX | right | 3 | -61 | -52 | 3 | 1.000 | 4.21 |
| 43 | VI | left | -16 | -66 | -20 | 16 | 1.000 | 4.17 |
| 44 | VIIIb | vermal | -1 | -63 | -48 | 1 | 1.000 | 4.15 |
| 45 | Crus I | left | -34 | -74 | -26 | 6 | 1.000 | 4.13 |
| 46 | Crus I | left | -31 | -59 | -34 | 3 | 1.000 | 4.12 |
| 47 | Crus I | right | 52 | -57 | -34 | 3 | 1.000 | 4.12 |
| 48 | Crus I | right | 34 | -67 | -40 | 4 | 1.000 | 4.09 |
| 49 | Crus I | left | -40 | -56 | -40 | 9 | 1.000 | 4.08 |
| 50 | Crus II | right | 28 | -70 | -40 | 7 | 1.000 | 4.08 |
| 51 | VIIb | right | 12 | -72 | -53 | 8 | 1.000 | 4.08 |
| 52 | VIIIa | right | 31 | -41 | -48 | 6 | 1.000 | 4.08 |
| 53 | Crus II | right | 17 | -80 | -35 | 5 | 1.000 | 4.07 |
| 54 | VI | right | 19 | -68 | -32 | 9 | 1.000 | 4.07 |
| 55 | Crus II | left | -25 | -68 | -40 | 4 | 1.000 | 4.06 |
| 56 | V | right | 4 | -59 | -24 | 5 | 1.000 | 4.05 |
| 57 | V | right | 29 | -35 | -30 | 2 | 1.000 | 4.04 |
| 58 | I-IV | right | 4 | -50 | -7 | 2 | 1.000 | 4.04 |
| 59 | V | left | -28 | -37 | -29 | 6 | 1.000 | 4.04 |
| 60 | Crus I | right | 49 | -53 | -40 | 4 | 1.000 | 4.01 |
| 61 | Crus II | left | -19 | -88 | -39 | 1 | 1.000 | 3.98 |
| 62 | Crus II | right | 38 | -73 | -48 | 3 | 1.000 | 3.97 |
| 63 | VI | right | 32 | -50 | -25 | 9 | 1.000 | 3.97 |
| 64 | Crus I | left | -42 | -55 | -40 | 1 | 1.000 | 3.95 |
| 65 | Crus II | left | -23 | -73 | -38 | 2 | 1.000 | 3.95 |
| 66 | VI | left | -27 | -65 | -31 | 3 | 1.000 | 3.94 |
| 67 | Crus I | right | 42 | -57 | -36 | 2 | 1.000 | 3.93 |
| 68 | I-IV | right | 17 | -37 | -19 | 3 | 1.000 | 3.92 |
| 69 | Crus II | right | 14 | -75 | -39 | 1 | 1.000 | 3.90 |
| 70 | Crus I | right | 52 | -59 | -33 | 3 | 1.000 | 3.88 |
| 71 | Crus I | left | -32 | -57 | -35 | 1 | 1.000 | 3.87 |
| 72 | Crus I | right | 36 | -72 | -36 | 3 | 1.000 | 3.86 |
| 73 | I-IV | left | -12 | -41 | -13 | 2 | 1.000 | 3.86 |
| 74 | V | right | 26 | -37 | -31 | 1 | 1.000 | 3.86 |
| 75 | Crus I | right | 42 | -59 | -29 | 1 | 1.000 | 3.86 |
| 76 | Crus II | left | -29 | -78 | -52 | 2 | 1.000 | 3.86 |
| 77 | Crus I | left | -47 | -51 | -39 | 1 | 1.000 | 3.84 |
| 78 | IX | vermal | -1 | -52 | -36 | 1 | 1.000 | 3.84 |
| 79 | Crus I | right | 37 | -51 | -33 | 1 | 1.000 | 3.83 |
| 80 | VIIIb | right | 24 | -40 | -51 | 2 | 1.000 | 3.83 |
| 81 | I-IV | left | -15 | -38 | -18 | 1 | 1.000 | 3.82 |
| 82 | I-IV | left | -13 | -40 | -15 | 1 | 1.000 | 3.81 |
| 83 | VI | left | -26 | -61 | -22 | 1 | 1.000 | 3.81 |
| 84 | Crus I | right | 41 | -64 | -38 | 2 | 1.000 | 3.81 |
| 85 | IX | left | -1 | -55 | -47 | 1 | 1.000 | 3.79 |
| *Note*. ^1^cluster size in voxel. | | | | | | | | |

| Table S13: Number of feedback-locked trials per condition for the MLM | | | | | | |  |  |
| --- | --- | --- | --- | --- | --- | --- | --- | --- |
| Immediate Feedback | |  |  | Delayed Feedback | | | |  |
| Learnable trials | | | | | | | | |
| Negative feedback high PE trials | | |  | Negative feedback high PE trials | | | | |
| patients | 328 |  |  | patients | | | 382 |  |
| controls | 387 |  |  | controls | | | 333 |  |
|  |  |  |  |  | | |  |  |
| Positive feedback high PE trials | | |  | Positive feedback high PE trials | | | | |
| patients | 377 |  |  | patients | | | 525 |  |
| controls | 573 |  |  | controls | | | 365 |  |
|  |  |  |  |  | | |  |  |
| Negative feedback low PE trials | | |  | Negative feedback low PE trials | | | | |
| patients | 793 |  |  | patients | | | 756 |  |
| controls | 888 |  |  | controls | | | 670 |  |
|  |  |  |  |  | | |  |  |
| Positive feedback low PE trials | | |  | Positive feedback low PE trials | | | | |
| patients | 1668 |  |  | patients | | | 1527 |  |
| controls | 1773 |  |  | controls | | | 2217 |  |
|  |  |  |  |  | | |  |  |
| Unlearnable trials | | | | | | | | |
| Negative feedback for high PE trials | | |  | Negative feedback for high PE trials | | | | |
| patients | 728 |  |  | patients | | | 952 |  |
| controls | 990 |  |  | controls | | | 1079 |  |
|  |  |  |  |  | | |  |  |
| Positive feedback high PE trials | | |  | Positive feedback high PE trials | | | | |
| patients | 868 |  |  | patients | | | 605 |  |
| controls | 780 |  |  | controls | | | 696 |  |
|  |  |  |  |  | | |  |  |
| Negative feedback for low PE trials | | |  | Negative feedback for low PE trials | | | | |
| patients | 943 |  |  | patients | | | 699 |  |
| controls | 906 |  |  | controls | | | 802 |  |
|  |  |  |  |  | | |  |  |
| Positive feedback low PE trials | | |  | Positive feedback low PE trials | | | | |
| patients | 630 |  |  | patients | | | 916 |  |
| controls | 960 |  |  | controls | | | 995 |  |
| *Note*. For the respective MLM after trial exclusion. | | | |  |  |  |  |  |

| Table S14. Trials per condition for the grand-averages | | | | | |  |
| --- | --- | --- | --- | --- | --- | --- |
| **Prediction Error** | **Valence** | **Timing** | ***M* Controls** | ***SD* Controls** | ***M* Patients** | ***SD* Patients** |
| high PEabs | negative | immediate | 15.5 | 12.3 | 15.6 | 8.7 |
| low PEabs | negative | immediate | 35.5 | 23.2 | 37.8 | 24.9 |
| high PEabs | positive | immediate | 22.9 | 20.4 | 18.9 | 15.6 |
| low PEabs | positive | immediate | 70.9 | 39.4 | 79.4 | 35.6 |
| high PEabs | negative | delay | 13.3 | 7.0 | 18.2 | 12.7 |
| low PEabs | negative | delay | 26.8 | 24.6 | 35.9 | 20.8 |
| high PEabs | positive | delay | 15.2 | 20.1 | 26.0 | 22.9 |
| low PEabs | positive | delay | 88.7 | 40.7 | 72.7 | 39.6 |
| *Note*. Mean = *M*, Standard deviation = *SD*. | | |  |  |  |  |

**References**

EuroQol, G. (1990). EuroQol--a new facility for the measurement of health-related quality of life. *Health Policy*, *16*(3), 199-208. <https://doi.org/10.1016/0168-8510(90)90421-9>

Faul, F., Erdfelder, E., Lang, A. G., & Buchner, A. (2007). G* Power 3: A flexible statistical power analysis program for the social, behavioral, and biomedical sciences. *Behavior research methods*, *39*(2), 175-191. https://doi.org/10.3758/BF03193146

Hobart, J. C., Riazi, A., Lamping, D. L., Fitzpatrick, R., & Thompson, A. J. (2003). Measuring the impact of MS on walking ability: the 12-Item MS Walking Scale (MSWS-12). *Neurology*, *60*(1), 31-36. <https://doi.org/10.1212/wnl.60.1.31>

Jacobi, H., Rakowicz, M., Rola, R., Fancellu, R., Mariotti, C., Charles, P., ... & Klockgether, T. (2013). Inventory of Non-Ataxia Signs (INAS): validation of a new clinical assessment instrument. The Cerebellum, 12, 418-428. <https://doi.org/10.1007/s12311-012-0421->3

Kroenke, K., Spitzer, R. L., & Williams, J. B. (2001). The PHQ-9: validity of a brief depression severity measure. *J Gen Intern Med*, *16*(9), 606-613. <https://doi.org/10.1046/j.1525-1497.2001.016009606.x>

Powell, L. E., & Myers, A. M. (1995). The Activities-specific Balance Confidence (ABC) Scale. *J Gerontol A Biol Sci Med Sci*, *50A*(1), M28-34. <https://doi.org/10.1093/gerona/50a.1.m28>

Rolls, E. T., Huang, C. C., Lin, C. P., Feng, J., & Joliot, M. (2020). Automated anatomical labelling atlas 3. *Neuroimage*, *206*, 116-189. https://doi.org/10.1016/j.neuroimage.2019.116189

Schmahmann, J. D., Gardner, R., MacMore, J., & Vangel, M. G. (2009). Development of a brief ataxia rating scale (BARS) based on a modified form of the ICARS. *Mov Disord*, *24*(12), 1820-1828. <https://doi.org/10.1002/mds.22681>

Schmitz-Hübsch, T., Giunti, P., Stephenson, D. A., Globas, C., Baliko, L., Sacca, F., Mariotti, C., Rakowicz, M., Szymanski, S., Infante, J., van de Warrenburg, B. P., Timmann, D., Fancellu, R., Rola, R., Depondt, C., Schols, L., Zdzienicka, E., Kang, J. S., Dohlinger, S., . . . Klockgether, T. (2008). SCA Functional Index: a useful compound performance measure for spinocerebellar ataxia. *Neurology*, *71*(7), 486-492. <https://doi.org/10.1212/01.wnl.0000324863.76290.19>

Trouillas, P., Takayanagi, T., Hallett, M., Currier, R. D., Subramony, S. H., Wessel, K., Bryer, A., Diener, H. C., Massaquoi, S., Gomez, C. M., Coutinho, P., Ben Hamida, M., Campanella, G., Filla, A., Schut, L., Timann, D., Honnorat, J., Nighoghossian, N., & Manyam, B. (1997). International Cooperative Ataxia Rating Scale for pharmacological assessment of the cerebellar syndrome. The Ataxia Neuropharmacology Committee of the World Federation of Neurology. *J Neurol Sci*, *145*(2), 205-211. <https://doi.org/10.1016/s0022-510x(96)00231-6>

Zigmond, A. S., & Snaith, R. P. (1983). The hospital anxiety and depression scale. *Acta Psychiatr Scand*, *67*(6), 361-370. <https://doi.org/10.1111/j.1600-0447.1983.tb09716.x>
